# The First-in-Human Whole-Body Dynamic Pharmacokinetics Study of Aptamer

**DOI:** 10.34133/research.0126

**Published:** 2023-05-09

**Authors:** Ding Ding, Haitao Zhao, Dali Wei, Qinglai Yang, Cai Yang, Ruowen Wang, Yumei Chen, Lianghua Li, Shuxian An, Qian Xia, Gang Huang, Jianjun Liu, Zeyu Xiao, Weihong Tan

**Affiliations:** ^1^ Institute of Molecular Medicine (IMM), Renji Hospital, State Key Laboratory of Oncogenes and Related Genes, Shanghai Jiao Tong University School of Medicine, and College of Chemistry and Chemical Engineering, Shanghai Jiao Tong University, Shanghai 200240, China.; ^2^ Department of Nuclear Medicine, Renji Hospital, School of Medicine, Shanghai Jiao Tong University, Shanghai 200127, China.; ^3^ Center for Molecular Imaging Probes, Cancer Research Institute, University of South China, Hengyang, Hunan 421001, China.; ^4^ The Key Laboratory of Zhejiang Province for Aptamers and Theranostics, Zhejiang Cancer Hospital, Hangzhou Institute of Medicine (HIM), Chinese Academy of Sciences, Hangzhou, Zhejiang 310022, China.; ^5^ Molecular Science and Biomedicine Laboratory (MBL), State Key Laboratory of Chemo/Biosensing and Chemometrics, College of Chemistry and Chemical Engineering, Hunan University, Changsha, Hunan 410082, China.; ^6^ Shanghai Key Laboratory of Molecular Imaging, Shanghai University of Medicine and Health Sciences, Shanghai 201318, China.; ^7^ Department of Pharmacology and Chemical Biology, Shanghai Jiao Tong University School of Medicine, Shanghai, 200127, China.

## Abstract

Serving as targeting ligands, aptamers have shown promise in precision medicine. However, the lack of knowledge of the biosafety and metabolism patterns in the human body largely impeded aptamers’ clinical translation. To bridge this gap, here we report the first-in-human pharmacokinetics study of protein tyrosine kinase 7 targeted SGC8 aptamer via in vivo PET tracking of gallium-68 (^68^Ga) radiolabeled aptamers. The specificity and binding affinity of a radiolabeled aptamer, named ^68^Ga[Ga]-NOTA-SGC8, were maintained as proven in vitro. Further preclinical biosafety and biodistribution evaluation confirmed that aptamers have no biotoxicity, potential mutation risks, or genotoxicity at high dosage (40 mg/kg). Based on this result, a first-in-human clinical trial was approved and carried out to evaluate the circulation and metabolism profiles, as well as biosafety, of the radiolabeled SGC8 aptamer in the human body. Taking advantage of the cutting-edge total-body PET, the aptamers’ distribution pattern in the human body was acquired in a dynamic fashion. This study revealed that radiolabeled aptamers are harmless to normal organs and most of them are accumulated in the kidney and cleared from the bladder via urine, which agrees with preclinical studies. Meanwhile, a physiologically based pharmacokinetic model of aptamer was developed, which could potentially predict therapeutic responses and plan personalized treatment strategies. This research studied the biosafety and dynamic pharmacokinetics of aptamers in the human body for the first time, as well as demonstrated the capability of novel molecular imaging fashion in drug development.

## Introduction

Nowadays, cancer treatment has entered a new era of precision medicine[1–3]. As an important part of precision medicine, nucleic acid has evolved from genetic materials to synthetic biological materials with various biological functions [4–7]. DNA nanotechnology and functional oligonucleotides such as DNAzyme and aptamer have been widely used in cancer molecular diagnostics and targeted therapy [8–10]. Serving as a unique targeting ligand, aptamers and aptamer-based theranostic platforms play a vital role in cancer precision medicine [11–13]. Aptamers are single-stranded nucleic acid molecules that fold into unique 3-dimensional structures with ligand-binding sites and interact with the desired target through complementarity in shape and charge [14]. Since aptamers are nucleic acids, they can be easily synthesized by chemical reactions, making them a kind of “chemical antibody” with binding affinity comparable to that of antibodies [15]. Aptamers possess several features such as easy generation, low in cost with low batch-to-batch variation, reversible folding properties, and low immunogenicity [16]. With the wide use of nucleic acid therapy, the financial cost of aptamer and aptamer-related biomedical applications will be greatly reduced. These merits facilitate their integration into bioanalysis, disease diagnostics, and targeted therapeutic applications in line with the objectives of precision medicine [17]. Among these applications, we highlight the conjugation of aptamers with anticancer therapeutic agents, i.e., aptamer–drug conjugates, or ApDCs. As the potential counterpart of antibody–drug conjugates, ApDCs selectively bind to its target overexpressed in tumors to deliver tumor-specific drugs. Through a simple chemical conjugation, aptamers are capable of delivering multiple kinds of cargos, including small interfering RNAs (siRNAs) [18], microRNAs (miRNAs) [19], and cytotoxic drugs [20]. Recently, triptolide-conjugated nucleolin targeting aptamer AS1411 demonstrated its efficacy in triple-negative breast cancer treatment [21]. Artesunate-conjugated aptamers have been successfully used in colorectal cancer treatment [22].

Although ApDCs show excellent therapeutic effects in preclinical studies, so far, very little is known about the pharmacokinetics of aptamers including absorption, distribution, metabolism, and excretion (ADME), which have a substantial impact on achieving desirable therapeutic effects of ApDCs in the human body. On the other hand, though few nucleic acid therapies such as antisense oligonucleotides and siRNAs have already been launched into the market with FDA approval, their preclinical evaluations are strict and more complicated than traditional drugs [23,24]. As a kind of novel therapeutic strategy, the preclinical evaluations such as biosafety and potential genotoxicity need to be rationally designed to fit into the nature of nucleic acid itself. The same issue also challenged the scenario of aptamers’ clinical translation. Therefore, to elucidate more comprehensively the biochemistry that allows aptamers to qualify for further clinical translation, we would need a technique that could collectively acquire the data suggested above via some in-depth, fast, whole-body, and dynamic imaging of aptamers’ biological fate. Positron emission tomography (PET) is widely recognized as the most sensitive means available for noninvasive tomographic imaging of physiologic, metabolic, and molecular pathways in living humans [25–27]. This technique scans the patient’s body and measures the photon emission of the administered radiotracer after positron decay for localization and quantification providing an in-depth 3D observation way of administrated radiotracer’s biological fate [28,29]. The possibility of using PET in drug development was demonstrated in neuroscience drug discovery for the quantitative assessment of new chemical entities’ exposure in the central nervous system [30]. Nevertheless, current PET scanners are not capable of studying whole-body drug pharmacokinetics because of the limited t axial field of view (FOV)[31,32]. Recently, the introduction of total-body PET (TB-PET) makes simultaneously acquiring static and dynamic 3D images of the entire human body technically feasible [33]. This major advance enables in vivo and real-time pharmacokinetic studies of radiolabeled drugs. On the other hand, the TB-PET enables continuous acquisition of the whole-body organs’ PET signals, which provides the possibility of developing a physiologically based pharmacokinetic (PBPK) model for humans. The PBPK modeling is substantially useful in precision medicine since this simulation provides the prediction of drugs’ pharmacokinetics in humans, including both intrinsic and extrinsic factors on ADME [34]. With the help of PBPK modeling, the therapeutic dosage of ApDCs for each patient could be personalized to achieve the best disease lesion drug delivery and minimal side effects on normal organs [35].

In this study, to investigate the biological fate of aptamers in the human body, the anti-PTK7 SGC8 aptamer was radiolabeled with a radioisotope ^68^Gallium via a chelator (2,2′,2′′-(1,4,7-triazacyclononane-1,4,7-triyl)triacetic acid, NOTA) and named ^68^Ga[Ga]-NOTA-SGC8. First, the radiolabeled SGC8 aptamer’s specific binding functionality was demonstrated to be reserved either in cells or in mouse xenografted tumor models. Second, a series of preclinical investigations were performed, including toxicity, biodistribution, and pharmacokinetics, to determine the biosafety of the aptamer for further clinical study. Third, a first-in-human phase 0 clinical study was conducted after approval to further investigate aptamers’ biosafety and pharmacokinetics in the human body in a comprehensive and dynamic fashion. With the help of TB-PET, a systemic study of aptamers’ biological fate was meticulously investigated in patients, and a PBPK modeling of aptamer was successfully established and the fitting results were close to the realistic data acquired from the clinic.

## Results

### Radiolabeling of aptamers and in vitro targeting functionality study

**Fig. 1.**
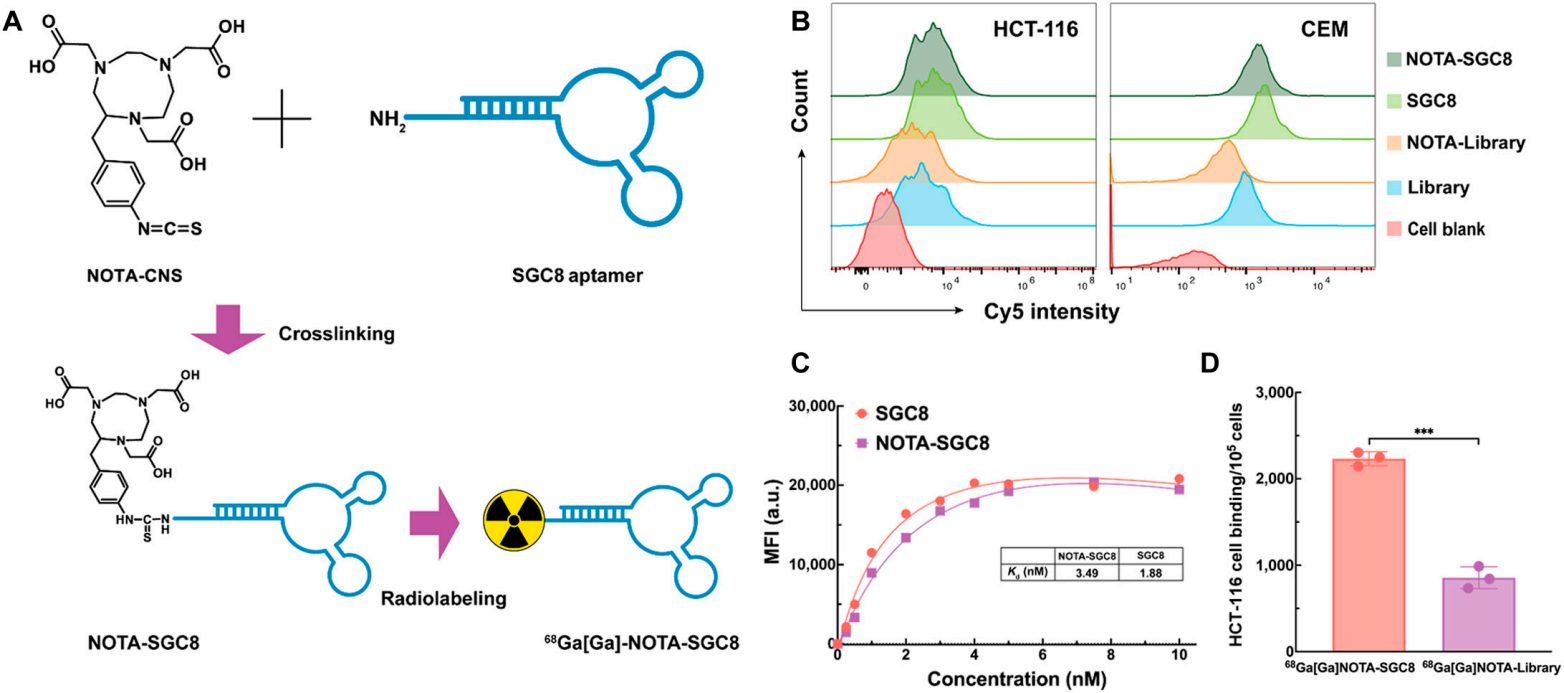
Radiolabeling of the SGC8 aptamer and targeting functionality investigation. (A) Schematic illustration of SGC8 aptamer radiolabeling. (B) Flow cytometry study of the SGC8 aptamer and NOTA-modified SGC8 aptamer specific binding on PTK-7-overexpressed cells. (C) Binding affinity study against target cells of NOTA-SGC8 and SGC8. *K*_d_ values are 3.49 nM and 1.88 nM, respectively. (D) Cell binding assay of radiolabeled aptamer ^68^Ga[Ga]-NOTA-SGC8 and ^68^Ga[Ga]-NOTA-Library, ***P ≤ 0.001. These results demonstrated that the SGC8 aptamer maintained its targeting functionality after NOTA modification and ^68^Ga radioisotope labeling.

NOTA-SGC8 (Fig. 1A) was synthesized by chemical coupling of the NH_2_-SGC8 aptamer and p-SCN-Bn-NOTA via an amide bond. The synthesis process includes solid-phase synthesis, ammonia deprotection, ion exchange column purification, desalting, lyophilization, coupling of NOTA molecules, reversed-phase column purification, etc. (Fig. S1). After purification, NOTA-SGC8 displayed greater than 95% ultra-performance liquid chromatography purity. Further identification of NOTA-SGC8 was confirmed by electrospray ionization mass spectrometry (Fig. S2A and B). The measured molecular weights of NH_2_-SGC8 and NOTA-SGC8 match the calculated molecular weights. For clinical use, NOTA-SGC8 was synthesized under GMP standards. Radioactive ^68^Ga was eluted from a ^68^Ge/^68^Ga generator, and ^68^Ga[Ga]-NOTA-SGC8 was readily prepared with greater than 98% radiolabeling yield and 16.7 MBq/mM specific activity. The final product was analyzed with reversed-phase high-performance liquid chromatography (RP-HPLC), and the retention time of ^68^Ga[Ga]-NOTA-SGC8 was 10.2 min (Fig. S2C and D).

To investigate the effects of NOTA conjugation and radiolabeling on the aptamer’s targeting capability, flow cytometry, binding affinity (*K*_d_), and γ-counter characterizations were performed. Since the aptamer’s targeting functionality is highly related to the secondary structure, circular dichroism spectrum characterization of the SGC8 aptamer and NOTA-SGC8 was studied and the secondary structure of aptamer was maintained (Fig. S3). The specific binding against protein tyrosine kinase 7 (PTK-7) receptor overexpressed HCT-116 and CEM cell lines was proven. As shown in the flow cytometry measurements (Fig. 1B), Cy5-labeled SGC8 and NOTA-SGC8 aptamers showed obvious fluorescence shifts compared with the control library group that demonstrated specific binding to HCT-116 and CEM cell lines. Meanwhile, as shown in Fig. 1C, the *K*_d_ values of SGC8 and NOTA-SGC8 were within the same order of magnitude, indicating that the chemical conjugation of SGC8 aptamers with NOTA did not affect the aptamer’s targeting abilities. The cell-binding assay of ^68^Ga[Ga]-NOTA-SGC8 against HCT-116 cells was also carried out, as shown in Fig. 1D. After incubation of ^68^Ga[Ga]-NOTA-SGC8 with HCT-116 cells, the radioactivity of each sample was measured with a γ-counter, and specific binding of HCT-116 cells was confirmed. In sum, NOTA modification and radiolabeling would not adversely affect the aptamers’ specific binding functionality.

### In vivo targeting functionality and pharmacokinetics study of radiolabeled aptamers

**Fig. 2.**
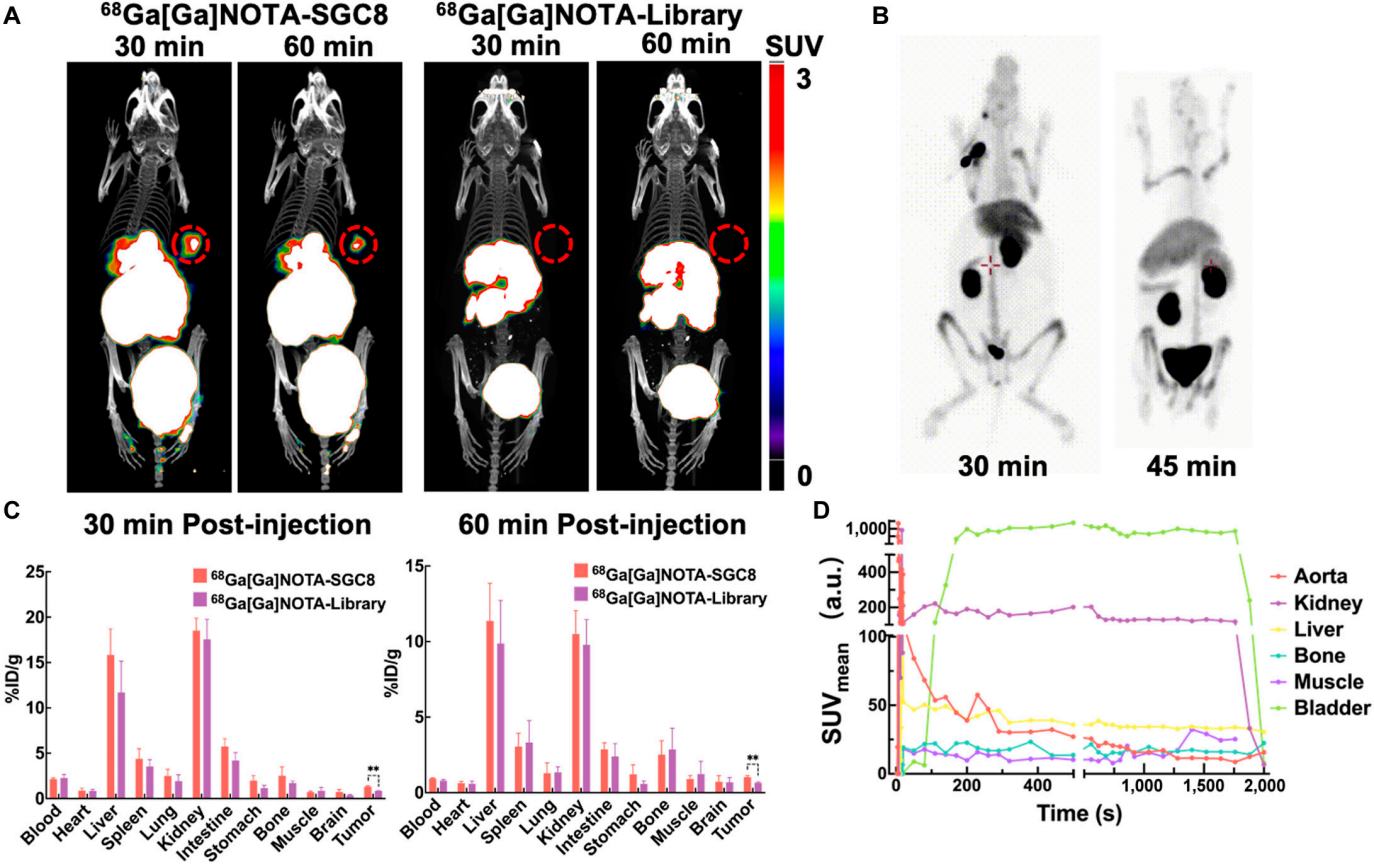
Radiolabeled SGC8 aptamer in vivo targeting functionality and pharmacokinetics investigations in different animal models. (A) Representative PET/CT imaging of HCT-116 human colon tumor-xenografted nude mice using ^68^Ga[Ga]-NOTA-SGC8 and ^68^Ga[Ga]-NOTA-Library as imaging agents at 30 and 60 min after i.v. administration. Tumor regions were labeled with red circles. (B) Pharmacokinetics of ^68^Ga[Ga]-NOTA-SGC8 on a rabbit model with PET imaging at 30 and 45 min after i.v. administration. (C) Biodistribution of ^68^Ga[Ga]-NOTA-SGC8 on HCT-116 human colon tumor-xenografted nude mice. The data are presented as percent injected dose/gram (%ID/g). Student’s *t* test was used for statistical comparison, *n* = 4, **P ≤ 0.01. (D) Quantitative time–radioactivity curves of ^68^Ga[Ga]-NOTA-SGC8 in rabbit’s major organs and tissues analyzed according to the quantification analysis of dynamic PET imaging over 0 to 30 min.

In vivo tumor imaging of ^68^Ga[Ga]-NOTA-SGC8 was studied with a small animal Micro-PET/CT system using a BALB/c nude mouse model with a PTK-7-overexpressed colorectal carcinoma (HCT-116 cell line) xenografted tumor. As shown in Fig. 2A, the static imaging of ^68^Ga[Ga]-NOTA-SGC8 and control ^68^Ga[Ga]-NOTA-Library was acquired at 0.5 and 1 h post-i.v. injection. Both groups possessed high liver, kidney, and bladder retention with a fairly clean background in other organs and tissues. Specific accumulation of ^68^Ga[Ga]-NOTA-SGC8 at tumor sites was observed at 0.5 and 1 h (Fig. 2A). Compared to the control library groups, ^68^Ga[Ga]-NOTA-SGC8 showed higher accumulation signals at tumor sites, confirming the radiolabeled aptamer’s specific binding functionality in vivo.

The biodistribution of ^68^Ga[Ga]-NOTA-SGC8 in the mice is depicted in Fig. 2C, and the result correlated well with the micro-PET/CT study. Tumor uptake of ^68^Ga[Ga]-NOTA-SGC8 and control library was 1.31 ± 0.09% and 0.84 ± 0.03% ID/g at 0.5 h after injection, respectively. One hour after injection, the tumor uptake of ^68^Ga[Ga]-NOTA-SGC8 and control library dropped to 1.03 ± 0.11% and 0.66 ± 0.05% ID/g. The accumulation of ^68^Ga[Ga]-NOTA-SGC8 in other normal organs was much lower than that in the liver and kidney. To further study the biodistribution of ^68^Ga[Ga]-NOTA-SGC8, a rabbit model was continuously imaged with dynamic PET/CT, as shown in Fig. 2B. Quantitative time–radioactivity curves of the aptamers in different organs were acquired, as shown in Fig. 2D. Similar to the results in the mouse model, the aptamers were rapidly redistributed after injection and cleared through the kidney, as data showed a fast signal increase at the bladder.

The in vitro stability of ^68^Ga[Ga]-NOTA-SGC8 was tested in phosphate buffer saline (PBS) and fetal bovine serum. As analyzed by HPLC (Fig. S4A), the radiolabeled aptamer was intact after 1 h of incubation. In vivo urine analysis revealed that 80% of the ^68^Ga[Ga]-NOTA-SGC8 excreted renally also remained intact in the collected urine from mice 1 h after injection. Blood half-life measurements on normal mice showed that the *t*_1/2_ of aptamers was 1.05 min (Fig. S4B). This result indicates that the aptamers might be fast cleared via metabolism organs because of the low molecular weights and water solubility. Further HPLC analysis of mouse blood at different time points post-injection showed that the radiolabeled aptamers could still be identified 15 min post-injection (Fig. S4C).

### Preclinical toxicity evaluation of aptamers

To evaluate the potential toxicity of the SGC8 aptamer, a single-dose toxicity test was designed using Sprague Dawley (SD) rats, as briefly summarized in Table. The connection between dosages and systemic reaction of SD rats was observed after intravenous administration of the SGC8 aptamer through the tail vein. The SD rats were given a single tail vein injection of 40 mg/kg and 80 mg/kg SGC8, which are 320- and 640-fold higher than the injection dosage of previous in vivo pharmacokinetics studies. After continuous observation for 14 days, all experimental animal groups with SGC8 aptamer administration were healthy with no abnormal symptoms and changes in body weight, food consumption, and hematological indicators (Figs. S5A and S6, Table S1). Therefore, the dosages designed for further clinical study are biosafe, since they are much lower than the safe dose in this toxicity test. Meanwhile, a mammalian erythrocyte micronucleus test was carried out to detect chromosomal damage or mitotic organ damage caused by the SGC8 aptamer in rat erythrocytes. The SD rats received tail vein administration of SGC8 aptamers with dosages similar to the prior toxicity study, and the body weight, hematological indicators (Fig. S5B and Table S2), or gross anatomy were also measured with no abnormal results. The bone marrow micronucleus test results are summarized in Supplementary Table S3. Noteworthy, in the high-dose SGC8 (80 mg/kg) group, the micronucleus rates were 3.71‰ and 4.63‰, which are statistically different from the normal saline group (*P* < 0.05). Though this result suggests that high-dose SGC8 (80 mg/kg) might damage the chromosomes or mitosis of rat erythrocytes and induce the formation of rat erythrocyte micronuclei, the low-dose (40 mg/kg) group is safe and still much higher than that of the designed dosages in the further clinical study.

**Table.** Summary of aptamer’s preclinical toxicity evaluation.

**Single-dose toxicity test**				
**Dosage**	**Clinical observation**	**Body weights**	**Hematology**	**Bone marrow micronucleus test**
40 mg/kg	Normal	Normal	Normal	Normal
80 mg/kg	Normal	Normal	Normal	Abnormal
*Normal/abnormal: Without/with statistical differences compared with the saline group				
**Mutagenic, carcinogenic, and genotoxicity test**				
**Salmonella typhimurium reverse mutation assay**				Negative
**Chromosomal aberration test in mammalian cells**				Negative

To further evaluate potential genotoxicity, an in vitro mammalian cell chromosome aberration test was performed (Fig. S7). The chromosome aberration rate of each SGC8 dose group did not increase greatly (0.33% ≤ aberration rate ≤ 3.00%), and no significant difference was seen in the chromosome aberration rate compared with the negative control group (*P* > 0.05). Therefore, it was determined that the SGC8 aptamer would not cause chromosomal aberrations in mammalian cells. Next, in order to evaluate the mutagenicity of the SGC8 aptamer and predict its genetic hazards and potential carcinogenic effects, a Salmonella typhimurium reverse mutation test was also performed (Fig. S8). TA97a, TA98, TA100, TA102, and TA1535 were selected as the test strains to detect whether the aptamer could cause bacterial reverse mutation. The average number of retrograde bacteria in each dose group was less than 2 times of that in the negative control group, and there was no dose-dependent increasing relationship, indicating that the SGC8 aptamers have no mutagenicity to the test strain. On the other hand, a cytotoxicity study of SGC8 and NOTA-SGC8 aptamers was carried out and the results also demonstrated their biosafety (Fig. S9). In sum, in these preclinical biosafety evaluation tests, the SGC8 aptamers would not generate single-dose toxicity, genotoxicity, and potential carcinogenic effects at the designed dosages for clinical studies.

### Patient enrollment and clinical study design

Based on the cumulative in vitro and in vivo preclinical evaluation described above, we conducted a single-arm, open-label, phase 0, first-in-human clinical trial to investigate the biosafety and dynamic pharmacokinetics of the intravenously administered SGC8 aptamer with ^68^Ga-radiolabeling. This study was posted in the Chinese Clinical Trial Registry (ChiCTR) (ChiCTR2000034507). Four patients were enrolled from July 2020 through June 2021. Patients’ characteristics are summarized in Table S4. According to the guidelines of Good Clinical Practice (Chinese National Medical Products Administration), all the enrolled patients had a diagnosis of cancer or medical history of cancer. A physical examination was carried out, including vital signs, routine hematuria, liver and kidney function, blood human chorionic gonadotropin (HCG) (female), and electrocardiogram. Adverse events before and after ^68^Ga[Ga]-NOTA-SGC8 PET/CT and TB-PET scans were recorded.

### First-in-human static and dynamic PET imaging of aptamers

**Fig. 3.**
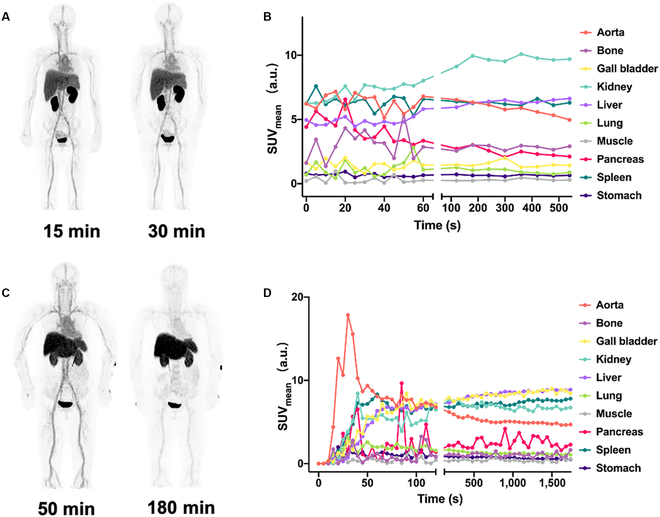
First-in-human PET imaging to evaluate the biodistribution and metabolism of radiolabeled SGC8 aptamers. Representative static PET imaging of 2 patients injected with ^68^Ga[Ga]-NOTA-SGC8. (A) Patient No. 1 was scanned at 15 and 30 min post-injection. (B) Patient No. 3 was scanned at 50 and 180 min post-injection. Quantitative time–radioactivity curves of patient No. 1 (C) and No. 3 (D) after ^68^Ga[Ga]-NOTA-SGC8 i.v. injection. SUV values in major organs and tissues analyzed according to dynamic PET/CT acquisition in 0 to 10 min and 0 to 30 min, respectively.

In-human PET/CT investigation of ^68^Ga[Ga]-NOTA-SGC8 biodistribution and metabolism was carried out with 4 patients. Patient No. 1, 2, and 3 were imaged with a traditional PET/CT system, and patient No. 4 was imaged with the uEXPLORER TB-PET. As shown in Fig. 3A, the ^68^Ga[Ga]-NOTA-SGC8 aptamer was rapidly distributed in the liver, and most of it accumulated in the kidney. Clearance via urine was visualized in the patient’s bladder at 15 min. At 30 min post-injection, the same result was confirmed. Dynamic acquisition of patients’ abdominal data is shown in Fig. 3B, and the signals were normalized to standard uptake value (SUV_B/W_). Like the static image results, the kidney showed the highest SUV and soon reaches a plateau, indicating that most of the radiolabeled aptamers were accumulated and cleared from it. Meanwhile, similar to preclinical studies, liver, and spleen also showed some degree of accumulation compared with other main organs. The same distribution pattern was also observed in patient No. 2. To study ^68^Ga[Ga]-NOTA-SGC8 biodistribution for a longer time, a static image of patient No. 3 was acquired at 50 and 180 min post-injection (Fig. 3C). After a long time circulation, ^68^Ga[Ga]-NOTA-SGC8’s signals were decreased at the aorta, but veins, liver, kidney, and spleen still showed high accumulations. As shown in Fig. 3D, the same tendencies of distribution and metabolism were confirmed in the abdominal dynamic acquisition of patient No. 3 within 30 min of aptamer administration.

**Fig. 4.**
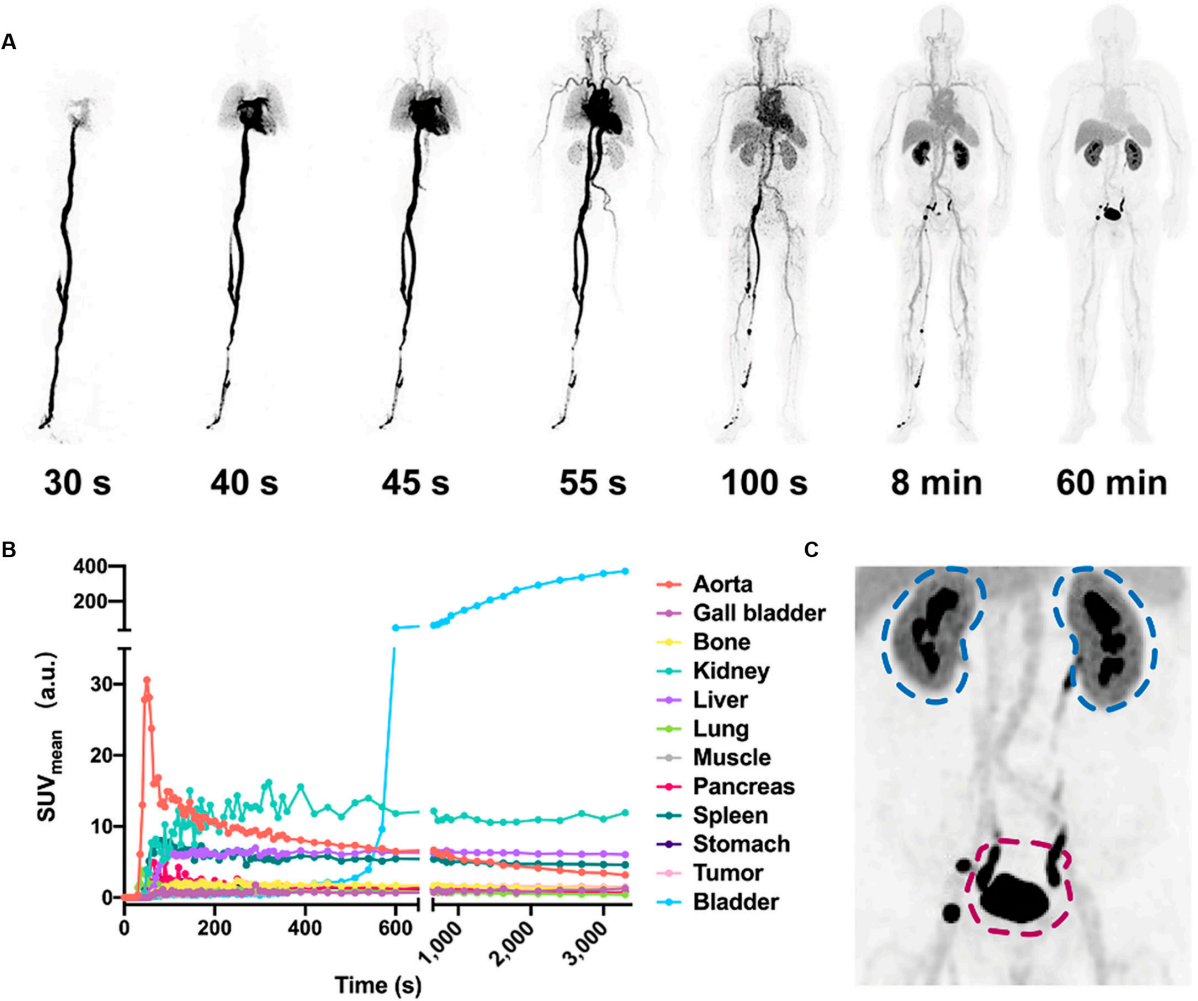
Whole-body dynamic PET imaging of radiolabeled SGC8 aptamers for pharmacokinetics study. (A) Whole-body dynamic imaging of the ^68^Ga[Ga]-NOTA-SGC8-injected patient at different time points post-administration, including 30, 40, 45, 55, and 100 s up to 60 min. (B) Quantitative time–radioactivity curves of major organs according to dynamic PET acquisition. (C) Amplified projection, including kidney (blue circle) and bladder (red circle). High-resolution biodistribution of ^68^Ga[Ga]-NOTA-SGC8 in kidney and bladder was visualized.

After a series of static and partial dynamic imaging, a PET/CT scan of patient No. 4 was carried out in the advanced uEXPLORER TB-PET system. As illustrated in Fig. 4A and Supplementary Movie S1, ^68^Ga[Ga]-NOTA-SGC8 circulated rapidly in the veins, resulting in aortic perfusion within 30 s. At 40 s, the aptamers went into lung vessels. Around 100 s post-injection, aptamers started accumulating in the liver, spleen, and kidney, and reached equilibrium around 8 min post-injection. The dynamic kinetic curves in Fig. 4B showed the same results. After rapid perfusion from aorta, aptamers accumulated in the liver, spleen and, kidney. The high-resolution image of the kidney and bladder in Fig. 4C demonstrated that the aptamers had cleared from kidney to bladder and accumulated in the kidney medulla. The bladder signal was dramatically increased around 8 min post-injection, indicating that the ^68^Ga[Ga]-NOTA-SGC8 started clearing from the kidney via urine and accumulated in the bladder. Aside from the study of ^68^Ga[Ga]-NOTA-SGC8, traditional ^18^F-FDG tracer was also applied to each patient for comparison (Fig. S10), and a totally different distribution pattern was observed.

### Whole-body PBPK modeling of aptamer

**Fig. 5.**
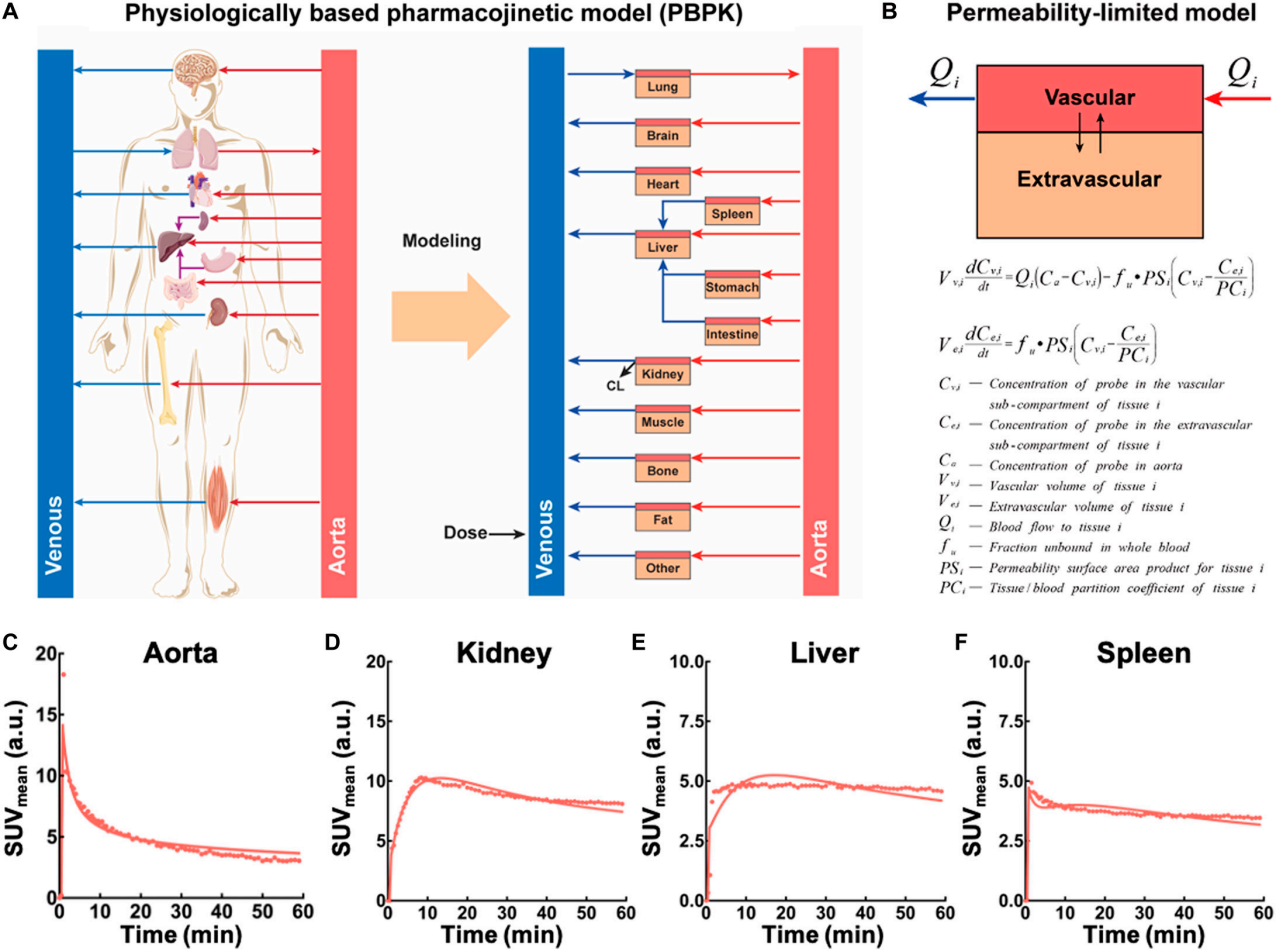
Development of aptamer’s whole-body PBPK modeling and fitting pharmacokinetics in the main metabolism organs. (A) Schematic illustration of the whole-body PBPK model for ^68^Ga[Ga]-NOTA-SGC8. The PBPK model consists of 14 compartments as depicted in the scheme. (B) The general permeability-limited model equations and parameters for the PBPK modeling. (C to F) Whole-body PBPK model fit pharmacokinetics profiles (line) versus observed data (dots) of (C) aorta, (D) kidney, (E) liver and (F) spleen.

The whole-body PBPK model of ^68^Ga[Ga]-NOTA-SGC8 was developed and fitted to the TB-PET continuously acquired patient’s data (Fig. 5A). A blood flow-driven, permeability-limited kinetics is employed to construct the PBPK model of aptamers (Fig. 5B). The best fit was generated with visual inspection and minimization of the mean squared error (MSE) between the simulated and the observed data. As depicted in Fig. 5C and Fig. S11, the simulated results including concentration-versus-time profiles were in good agreement with the clinically measured data in each compartment. It is worthy to point out that in the first few minutes post-injection, the measured data showed significant discrepancies from the model predictions, which is mainly due to the inhomogeneous dispersion of the aptamers. Besides, since the accumulation of ^68^Ga[Ga]-NOTA-SGC8 in the gallbladder was markedly lower than the bladder, hepatic excretion was ignored in the modeling process. All the estimated parameters required for PBPK modeling are listed in Table S5. Compared to other organs, the higher tissue/blood partition coefficient of the kidney, liver, and spleen indicates higher accumulations of ^68^Ga[Ga]-NOTA-SGC8 in these organs, which are coincident with that of the clinically acquired patient’s data.

## Discussion

Although aptamer-based biomedical applications have demonstrated promise in preclinical studies, clinically used aptamer-related drugs and registered clinical trials are still rare. Presently, most aptamer-related clinical trials are associated with Macugen (Pegaptanib), which is designed for age-related macular degeneration treatment [36]. Only a few aptamers are designed for intravenous administration, such as ARC1779 (NCT00632242) for the treatment of platelet function disorders[37], NOX-A12/Keytruda (NCT03168139) for colorectal and pancreatic cancer treatments [38], and the ^68^Ga-labeled SGC8 aptamer (NCT03385148) for imaging colorectal cancer patients [39]. While the translational value of aptamers is promising, the nature of nucleic acid makes it difficult to establish their biosafety and pharmacokinetic evaluations in the human body [40]. Total-body PET, one of the most advanced medical imaging techniques yet, scans a patient’s whole body with high-resolution and real-time imaging [41]. As a cutting-edge drug development method, this technique offers a new choice to evaluate the pharmacokinetics of aptamers in a whole-body dynamic fashion.

Before carrying out the clinical trial of aptamer biosafety and whole-body dynamic pharmacokinetics, it is essential to meticulously study its general toxicity, cytotoxicity, and genotoxicity. In the SD rats’ model single-dose toxicity evaluation, the dosages of SGC8 aptamers (40 and 80 mg/kg) are proven to be safe, which are much higher than the dosage in the following clinical study design. Meanwhile, the bone marrow micronucleus analysis suggests that SGC8 (40 mg/kg) would not damage the chromosomes or mitosis of rat erythrocytes and induce the formation of rat erythrocyte micronuclei. Further testing evaluated the mutagenicity of the SGC8 aptamer, as well as predicted its genetic hazards and potential carcinogenic effects. According to the results of these studies, the SGC8 aptamer showed neither mutagenicity nor genetic toxicity under the test conditions. These preclinical biosafety evaluations laid a solid foundation for further clinical evaluation of the aptamer’s biosafety and pharmacokinetics.

In this first-in-human clinical study, a total of 4 patients were enrolled. ^68^Ga[Ga]-NOTA-SGC8 PET/CT scan illustrated the biodistribution of aptamers in the human body for the first time. In the study of the first 3 patients, images with good quality were achieved, and aptamers were mainly cleared through the liver and kidney, coincident with results of the preclinical studies. Unlike the traditional clinically used ^18^F-FDG tracer (Fig. S10), which is characterized by considerable brain and intestine accumulation, aptamers were mainly distributed in the liver, spleen, kidney, and bladder, while the uptake of other organs was low. This biodistribution pattern of aptamers indicates that aptamer-based PET imaging agents would have better signal background control, allowing the acquisition of high signal/noise ratio images. In the further investigation of patient No. 4, the advanced TB-PET system was used to achieve detailed and high-quality whole-body dynamic images. As shown in Fig. 4 and Movie S1, the entire distribution process of radiolabeled aptamers was recorded dynamically from injection to redistribution in different organs. Radiolabeled aptamers are clearly shown to move from foot vein to aorta perfusion, and all movements along with blood flow and final accumulation in different organs were imaged with high quality. The metabolism fate of aptamers was dynamically recorded in the human body for the first time. Most of the radiolabeled aptamers were filtered through the kidney and accumulated in the bladder. Compared with traditional static images acquired from either PET or fluorescence imaging, TB-PET exhibits the whole metabolism behaviors of aptamers rather than the speculation based on the location of aptamers in different organs. Meanwhile, it also revealed that the accumulation of aptamers in the liver is slower than kidney, which is hard to observe in the static imaging modality. Notably, the high-resolution projection of kidney indicated that aside from the clearance via urine, a small portion of aptamers was accumulated in the renal pelvis. This explains the results in the preclinical study in which most aptamers were accumulated in the kidney, but not fully cleared via urine.

In the past, it was difficult for PBPK modeling to make quantitative pharmacokinetic estimations and predictions in drug development and precision medicine due to the requirements of modern computational power and inadequate data from animal studies. Taking advantage of the TB-PET extraordinary data collecting capacity, all the organs of the human body could be dynamically and simultaneously scanned, providing the possibility of developing a PBPK model close to the realistic aptamer’s pharmacokinetics. In this work, the developed PBPK model predicted the pharmacokinetics of aptamers in good agreement with clinically acquired data. The successful construction of the PBPK model based on the TB-PET acquired data offered a useful tool for more efficient disease diagnosis and lesion detection by comparing pharmacokinetic variations between patients and healthy individuals or lesion sites and healthy sites using PBPK modeling. Compared to the conventional compartment models, the PBPK modeling allows the prediction of the effects of various physiological and pathological parameters as well as multiple properties of the aptamers on the pharmacokinetic process by mathematical modeling, which can help individualize drug dosage estimation, even for special populations (e.g., children, the elderly, and people with renal insufficiency), and facilitate the development of human-friendly aptamers and ApDCs. Thanks to the PBPK modeling, personalized treatment plans could be rationally designed with the best drug delivery efficacy and minimal side effect on normal organs.

In conclusion, we have systemically studied the radiolabeled aptamers’ pharmacokinetics in the human body for the first time. Beyond the traditional pharmacokinetic evaluations that are typically done with animal models, PET imaging was used to realize the in situ visualization of aptamer biodistribution. Moreover, the introduction of advanced TB-PET allowed the dynamical whole-body signal acquisition of the human being. Based on the tremendous data acquired from TB-PET, the realistic pharmacokinetics in the human body of radiolabeled aptamers was evaluated. The clearance process of aptamers through the kidney via urine was demonstrated in a dynamic fashion. Meanwhile, taking advantage of the TB-PET continuously acquired data, a PBPK model close to the realistic aptamer pharmacokinetics was established, which could potentially be used for future ApDC therapeutic response prediction. On the other hand, the targeting and specificity of ^68^Ga[Ga]-NOTA-SGC8 were demonstrated in vitro and in vivo. An in-depth preclinical biosafety investigation, including general toxicity, cytotoxicity, and genotoxicity, was conducted. All of these efforts proved the potential of ^68^Ga[Ga]-NOTA-SGC8 to be used in PTK-7-positive tumor PET imaging and inspired us to develop more aptamer-based PET imaging agents in the future. In sum, this study not only laid a solid foundation for aptamers but also included nucleic acid-related medical clinical translation and demonstrated the feasibility of novel drug development fashion under the guidance of TB-PET at the same time.

## Materials and Methods

### Study design

This study aimed to evaluate the safety and dynamic pharmacokinetics of aptamer SGC8 in the human body. Toxicology was determined in preclinical tests. This clinical trial was a prospective, open-label, single-center, single-arm phase 0 first-in-human trial to determine the biosafety and pharmacokinetics of intravenously administered radiolabeled SGC8 aptamer in patients. This investigator-initiated clinical trial was conceived, developed, and performed in the Nuclear Department, Renji Hospital, Shanghai Jiao Tong University School of Medicine. The study was conducted under Renji Hospital Ethics Committee approval.

### SGC8 aptamer synthesis and radiochemistry

HPLC-purified SGC8 aptamer and control library sequences with fluorescence labeling were synthesized by Sangon Biotech, Shanghai, China, and Biosyntech, Soochow, China. SGC8 aptamer: 5′-ATC TAA CTG CTG CGC CGC CGG GAA AAT ACT GTA CGG TTA GA-3′. Library sequence: 5′-ATC TAA CTG ANN NNN NNN NNN NNN NNN NNN NNN CGG TTA GA-3′. N refers to random bases. The purity of all DNA sequences was detected by HPLC, and the molecular weights were confirmed by mass spectrum. Aptamers used for flow cytometry were labeled with a Cy5 dye at the 5′-end. NOTA-SGC8 was synthesized by conjugation of p-SCN-Bn-NOTA with 5′ amino-modified SGC8. Final products were characterized by mass spectrometry and HPLC. Clinical trial using NOTA-SGC8 was prepared under Good Manufacture Practice of Medical Products (GMP) by SynTheAll Pharmaceutical, Shanghai, China. For radiolabeling, ^68^GaCl_3_ solution (2 ml) was acquired from the ^68^Ge/^68^Ga generator with 0.1 M HCl elution. Next, NOTA-SGC8 (100 μg) precursors were dissolved in sodium acetate aqueous buffer (1 M, 300 μl) and mixed with ^68^GaCl_3_ solution. The mixture was allowed to react for 30 min at 90°C in a metal incubator. After cooling down to room temperature, the solution was purified with a NAP-5 column (GE Healthcare) to obtain ^68^Ga[Ga]-NOTA-SGC8 saline solution. The radiochemistry purity and stability of ^68^Ga[Ga]-NOTA-SGC8 were measured by HPLC.

### In vitro targeting functionality study of radiolabeled aptamer

HCT-116 cells were harvested by 0.25% EDTA digestion and washed with washing buffer (D-PBS supplemented with 4.5 g/L of glucose and 5 mM of MgCl_2_) via centrifugation. The harvested cells were counted and aliquoted to 300,000 cells in 200 μl of binding buffer. CEM suspension cells were treated like HCT-116 cells, but without EDTA digestion. Then, either SGC8 aptamer or library sequence was added to these cell aliquots at a final concentration of 250 nM. All samples were incubated on ice for 30 min. Flow cytometry was performed on a BD FACS flow cytometer. Median value was used here to avoid the interference of extreme values. All samples were tested in triplicate. To investigate the dissociation constant (*K*_d_) of the NOTA-SGC8 aptamer, the same treatment was performed on HCT-116 cells and incubated with various concentrations of aptamers. The *K*_d_ values of aptamers on HCT-116 cells were calculated by fitting the dependence of each sample’s median fluorescence intensity on the aptamer’s concentration by using GraphPad Prism.

A ^68^Ga[Ga]-NOTA-SGC8 cell-binding assay was also carried out. HCT-116 cells were harvested and aliquoted to 1,000,000 in each tube. ^68^Ga[Ga]-NOTA-SGC8 solution was added to each of the 2 tubes. After incubating at 4 °C for 4 h, 0.5 ml of the supernatant was collected after vigorous centrifugation. The radioactivity of both samples was measured using a γ-counter (2470 Wizard2, Perkin Elmer, Massachusetts, USA).

### In vivo targeting functionality and biodistribution studies of the radiolabeled aptamer

In vivo PET/CT imaging of mice was acquired by using the MicroPET/CT system (IRIS PET/CT, Inviscan, Strasbourg, France). Briefly, tumor-bearing mice were intravenously injected with 3.7 MBq of ^68^Ga[Ga]-NOTA-SGC8 and ^68^Ga[Ga]-NOTA-Library and anesthetized through inhalation of 2% isoflurane. Static imaging was acquired at 0.5 and 1 h post-injection, and dynamic images at 1 h post-injection were also acquired. Micro PET/CT imaging data were reconstructed with Monte Carlo-based 3-dimensional ordered subset expectation maximization (Monte Carlo-based 3D OSEM). Images and regions of interest were processed using Osirix MD software. An experimental rabbit PET/CT study was carried out by ear-marginal vein administration of ^68^Ga[Ga]-NOTA-SGC8 with a dosage of 0.05 mCi/kg. Rabbits were imaged using a United Imaging 780 PET/CT system with 2% pentobarbital sodium anesthesia. CT scanning and reconstruction parameters were as follows: 120 kV, automatic milliampere, slice thickness 3.0 mm, slice spacing 1.5 mm, and matrix 512 × 512. PET scanning and reconstruction parameters: number of iterations 3, subset 20, image size 192×192, layer thickness 2.68, smoothness Smooth2, Gaussian half-height width 3.0, and FOV 600 mm.

To evaluate the distribution of radiolabeled aptamers in tumor tissues and major organs, female nude mice with subcutaneous HCT-116 tumor xenografts were injected with 0.37 MBq of ^68^Ga[Ga]-NOTA-SGC8 and ^68^Ga[Ga]-NOTA-Library, respectively (*n* = 4 per group). Mice were sacrificed and dissected at 0.5 and 1 h post-injection. Blood, tumor, major normal tissues, and organs were collected and weighed, and their radioactivity was measured by a γ-counter. The results are presented as percentage of injected dose per gram of tissue (%ID/g).

### ^68^Ga[Ga]-NOTA-SGC8 physiological stability and pharmacokinetics studies

In vitro stability of ^68^Ga[Ga]-NOTA-SGC8 was evaluated in PBS and FBS. Briefly, equal volumes of ^68^Ga[Ga]-NOTA-SGC8 and PBS/FBS were mixed and incubated at room temperature for 1 h. Then, the radiochemical purity of samples was analyzed with HPLC equipped with a radioactive detector. In vivo stability and plasma half-life were evaluated on mice. ^68^Ga[Ga]-NOTA-SGC8 was administered to mice through tail vein injection at a dosage of 300 μCi. Blood and urine were collected from mice at 1 h post-injection, and samples were centrifuged and filtered for HPLC analysis. The assays were repeated twice.

### First-in-human PET imaging and pharmacokinetics studies of the radiolabeled aptamer

This study began in July of 2020 and ended in June of 2021. A total of 4 patients were enrolled, each providing written informed consent in accordance with institutional guidelines during the screening period. Patients were required to have a diagnosis of cancer or medical history of cancer. A physical examination was carried out, including vital signs, routine hematuria, liver and kidney function, blood HCG (female), and electrocardiogram, all completed within 1 week of PET/CT inspection. Static and dynamic images of patient nos. 1, 2, and 3 were acquired on a United Imaging uMI780 system with default injection dose 3 mCi. Patient No. 4 was scanned with the uEXPLORER TB-PET system with default injection dose 6 mCi. Name, gender, age, PET number, and weight of patients were double checked before ^68^Ga[Ga]-NOTA-SGC8 administration. ^68^Ga[Ga]-NOTA-SGC8 was administered via a bedside intravenous injection, and patients were instructed to empty the bladder as much as possible about 3 min before injection. Patients’ blood and urine samples were collected after PET/CT scans. Dynamic imaging acquisition was started with the injection of ^68^Ga[Ga]-NOTA-SGC8 at the same time. The collection method was dynamic, and the time was on demand. Static imaging was started with a CT scan to attenuate correction of the later PET scan, ranging from the top of the head to the knees. Lateral PET scan acquisition time was 2 min/slice, with a longer imaging time (90 min post-injection) of 3 min/slice. Practical injection dose and time were recorded after PET image acquisition. CT scan and reconstruction parameters were as follows: 120 kV for the whole body with automatic mA. Layer thickness was 3.0 mm, FOV was 500 mm, and reconstruction was Abdomen, B_SOFT_B. PET scanning and reconstruction parameters were as follows: whole-body acquisition 2 min per bed, the reconstruction parameter was OSEM, number of iterations was 3, subset was 20, image size was 192×192, smoothness was Smooth 3, and Gaussian half-height width was 3.0. In the uEXPLORER TB-PET scan, CT scan and reconstruction parameters were as follows: 120 kV for the whole body with automatic mA, slice interval was 1.5 mm, number of images was 1,305, matrix was 512 × 512, FOV was 500 mm, filter function B_SOFT_B, reconstruction parameter was KARL 3D. PET scan and reconstruction parameters were as follows: the number of iterations was 3, the subset was 20, the image size was 192 × 192, the layer thickness was 2.886, the smoothness was Smooth 2, the Gaussian width at half maximum was 3.0, and the FOV was 600 mm. PET dynamic framing was as follows: 36 × 5 s, 18 × 10 s, 8 × 30 s, 5 × 60 s, 5 × 180 s, and 6 × 300 s, a total of 78 frames. Physical examination was carried out, including vital signs, routine hematuria, liver and kidney function, and electrocardiogram. Adverse events were recorded on the next day of PET scan.

### Whole-body PBPK modeling of aptamer

A whole-body PBPK model was developed to investigate the pharmacokinetic behavior of ^68^Ga[Ga]-NOTA-SGC8 in humans, using Simbiology/MATLAB (MATLAB R2021a, The MathWorks, USA). The model consists of 14 compartments: aorta, venous, lung, brain, heart, liver, spleen, stomach, intestine, kidney, muscle, bone, fat, and other tissues; each tissue compartment is further divided into a vascular and an extravascular sub-compartment. It is assumed that the distribution process of the probe between these compartments is driven by blood flow, and the permeability-limited kinetics is employed to characterize the transfer of probes between the vascular and extravascular sub-compartments (Eqs. S1 to S6). The physiological parameters of the model were taken from the PK-Sim (Open Systems Pharmacology Suite Version 10.0) (SI Appendix, Table S6). The values of the permeability surface area product (*PS_i_*) represented the passive diffusion between the 2 sub-compartments, and the tissue/blood partition coefficients (*PC_i_*), the fraction of unbound probe in whole blood (*f_u_*), and the renal clearance (*CL_renal_*) were estimated using the maximum likelihood estimation method. For model fit and parameter estimation, the volumes of interest (VOIs) were delineated based on the fused PET/CT image of patient No. 4 to obtain the SUV_B/W_ of each compartment.

### Statistical analysis

Data processing and statistical analysis were performed using Excel (Microsoft) and GraphPad Prism (GraphPad Software LLC.). Student’s *t* test was applied for unpaired data, and a 95% confidence level was chosen to determine the significant difference between groups in cellular binding and tumor uptake of ^68^Ga[Ga]-NOTA-SGC8. The differences at the 95% confidence level (*P* < 0.05) were considered significant.

## Data Availability

All data associated with this study are present in the paper. Request for further information will be handled by the corresponding authors.

## Supplementary Materials

Supplementary 1 Materials and Methods Fig. S1. Synthesis route of the NOTA-SGC8 aptamer. Fig. S2. Mass and HPLC analysis of NOTA-SGC8 and radiolabeled SGC8 aptamers. Fig. S3. Circular dichroism spectrum characterization of SGC8 and NOTA-SGC8 aptamers. Fig. S4. Physiological stability and pharmacokinetics investigations of ^68^Ga[Ga]-NOTA-SGC8. Fig. S5. Body weight measurements of SD rats in the single-dose toxicity and mammalian erythrocyte micronucleus test. Fig. S6. Food consumption measurements of SD rats in the single-dose toxicity study. Fig. S7. Mammalian cell chromosome aberration test of the SGC8 aptamer for genotoxicity evaluation. Fig. S8. Salmonella typhimurium reverse mutation test of the SGC8 aptamer for mutagenicity evaluation. Fig. S9. Cytotoxicity investigation of SGC8 and NOTA-SGC8 aptamers. Fig. S10. ^68^Ga[Ga]-NOTA-SGC8 and ^18^F-FDG PET/CT imaging of patient No. 4. Fig. S11. Whole-body PBPK model fit pharmacokinetic profiles versus observed data of other tissues. Table S1. Hematology analysis of the SD rats in the single-dose toxicity investigation. Table S2. Hematology analysis of the SD rats in the mammalian erythrocyte micronucleus test. Table S3. Genotoxicity analysis of SGC8 aptamers by the bone marrow micronucleus test in the SD rats. Table. S4. Patient characteristics in the aptamer first-in-human dynamic pharmacokinetics study. Table S5. Human physiological parameters used to build the whole-body PBPK model and the delineated methods of each VOI. Table S6. PBPK model estimated parameters. Eqs. S1 to S6. Ordinary differential equations of the whole-body PBPK model.

Supplementary 2 Movie S1. Whole-body PET/CT dynamic scan of ^68^Ga[Ga]-NOTA-SGC8 administered to patients.
