# Supplementary material for: The First-in-Human Whole-Body Dynamic Pharmacokinetics Study of Aptamer
Source: Research (Wash D C). 2023 May 9;6:0126. doi: 10.34133/research.0126 (PMC10202413; doi:10.34133/research.0126)
Supplement: Supplementary 1 — Materials and Methods Fig. S1. Synthesis route of the NOTA-SGC8 aptamer. Fig. S2. Mass and HPLC analysis of NOTA-SGC8 and radiolabeled SGC8 aptamers. Fig. S3. Circular dichroism spectrum characterization of SGC8 and NOTA-SGC8 aptamers. Fig. S4. Physiological stability and pharmacokinetics investigations of 68Ga[Ga]-NOTA-SGC8. Fig. S5. Body weight measurements of SD rats in the single-dose toxicity and mammalian erythrocyte micronucleus test. Fig. S6. Food consumption measurements of SD rats in the single-dose toxicity study. Fig. S7. Mammalian cell chromosome aberration test of the SGC8 aptamer for genotoxicity evaluation. Fig. S8. Salmonella typhimurium reverse mutation test of the SGC8 aptamer for mutagenicity evaluation. Fig. S9. Cytotoxicity investigation of SGC8 and NOTA-SGC8 aptamers. Fig. S10. 68Ga[Ga]-NOTA-SGC8 and 18F-FDG PET/CT imaging of patient No. 4. Fig. S11. Whole-body PBPK model fit pharmacokinetic profiles versus observed data of other tissues. Table S1. Hematology analysis of the SD rats in the single-dose toxicity investigation. Table S2. Hematology analysis of the SD rats in the mammalian erythrocyte micronucleus test. Table S3. Genotoxicity analysis of SGC8 aptamers by the bone marrow micronucleus test in the SD rats. Table. S4. Patient characteristics in the aptamer first-in-human dynamic pharmacokinetics study. Table S5. Human physiological parameters used to build the whole-body PBPK model and the delineated methods of each VOI. Table S6. PBPK model estimated parameters. Eqs. S1 to S6. Ordinary differential equations of the whole-body PBPK model. [file research.0126.f1.docx]

The First-in-human Dynamical Pharmacokinetics Study of Aptamer

Ding Ding^1†^, Haitao Zhao^2†^, Dali Wei^1^, Qinglai Yang^1,3^, Cai Yang^1,4,5^, Ruowen Wang^1^, Yumei Chen^2^, Lianghua Li^2^, Shuxian An^2^, Qian Xia^1,2^, Gang Huang^2,6^, Jianjun Liu^2*^, Zeyu Xiao^1,7*^, Weihong Tan^1,4,5*^

**Affiliations:**

^1^Institute of Molecular Medicine (IMM), Renji Hospital, State Key Laboratory of Oncogenes and Related Genes, Shanghai Jiao Tong University School of Medicine, and College of Chemistry and Chemical Engineering, Shanghai Jiao Tong University, Shanghai 200240, China.

^2^Department of Nuclear Medicine, Renji Hospital, School of Medicine, Shanghai Jiao Tong University, Shanghai 200127, China.

^3^Center for Molecular Imaging Probes, Cancer Research Institute, University of South China, Hengyang, Hunan 421001, China.

^4^The Cancer Hospital of the University of Chinese Academy of Sciences (Zhejiang Cancer Hospital), Hangzhou Institute of Medicine (HIM), Chinese Academy of Sciences, Hangzhou, Zhejiang 310022, China.

^5^Molecular Science and Biomedicine Laboratory (MBL), State Key Laboratory of Chemo/Biosensing and Chemometrics, College of Chemistry and Chemical Engineering, Hunan University, Changsha, Hunan 410082, China.

^6^Shanghai Key Laboratory of Molecular Imaging, Shanghai University of Medicine and Health Sciences, Shanghai 201318, China.

^7^Department of Pharmacology and Chemical Biology, Shanghai Jiao Tong University School of Medicine, Shanghai, 200127, China.

*Corresponding authors:

Zeyu Xiao, Email: zxiao@sjtu.edu.cn

Jianjun Liu, Email: nuclearj@163.com

Weihong Tan, Email: tan@hnu.edu.cn

**Table of contents**

Supplementary Methods

Fig. S1. Synthesis route of NOTA-SGC8 aptamer.

Fig. S2. Mass and HPLC analysis of NOTA-SGC8 and radiolabeled SGC8 aptamer.

Fig. S3. Circular dichroism spectrum characterization of SGC8 and NOTA-SGC8 aptamer.

Fig. S4. Physiological stability and pharmacokinetics investigations of ^68^Ga[Ga]-NOTA-SGC8.

Fig. S5. Bodyweights measurements of SD rats in single-dose toxicity and mammalian erythrocyte micronucleus test.

Fig. S6. Food consumption measurements of SD rats in single-dose toxicity study.

Fig. S7. Mammalian cell chromosome aberration test of SGC8 aptamer for genotoxicity evaluation.

Fig. S8. Salmonella typhimurium reverse mutation test of SGC8 aptamer for mutagenicity evaluation.

Fig. S9. Cytotoxicity investigation of SGC8 and NOTA-SGC8 aptamer.

Fig. S10. ^68^Ga[Ga]-NOTA-SGC8 and ^18^F-FDG PET/CT imaging of patient No.4.

Fig. S11. Whole-body PBPK model fit PK profiles versus observed data of other tissues.

Table. S1. Hematology analysis of the SD rats in the single-dose toxicity investigation.

Table. S2. Hematology analysis of the SD rats in the mammalian erythrocyte micronucleus test.

Table. S3. Genotoxicity analysis of SGC8 aptamers by bone marrow micronucleus test in the SD rats.

Table. S4. Patient characteristics in aptamer first-in-human dynamic Pharmacokinetics study.

Table. S5. Human physiological parameters used to build the whole-body PBPK model and the delineated methods of each VOI.

Table. S6. PBPK model estimated parameters.

Eq. S1-S6 Ordinary Differential Equations of whole-body PBPK model.

**Supplementary Methods**

**Characteristics of aptameric structures**

The secondary structures of SGC8 and NOTA-SGC8 aptamer was studied by circular dichroism (CD) spectrum on Chirascan V100 and nitrogen protection was maintained throughout the experimental. All the aptamers were diluted with DPBS supplemented with 5 mM of MgCl_2_. All samples were diluted to 1 µM and scanned from 320 to 200 nm in a customized quartz cuvette. All samples underwent annealing treatments before detection.

**Preclinical toxicokinetic and toxicology studies**

A single-dose toxicity study and bone marrow micronucleus testing were designed with SD rats for aptamer toxicity evaluation. In total, 36 SD rats (SPF grade), half male and half female, were randomly divided into 6 groups on average. Animals in groups 1-3 were used for general toxicity studies. Each group consisted of 3 animals of each sex, namely the saline group (0 mg/kg) and the SGC8 low- and high-dose groups (40 mg/kg and 80 mg/kg, respectively). Animals in groups 4-6 were used for bone marrow micronucleus testing, and grouping was consistent with that in the single-dose toxicity testing. All the animals were administered by tail vein injection, and the administration volume was 5 mL/kg. The animals in the general toxicity test group were administered once for 14 consecutive days; the animals in the bone marrow micronucleus test group were administered once a day for 2 consecutive days with an interval of 24±1 hours. Before administration, a comprehensive physical examination of all animals was conducted. During the experiment, except on the day when the animals were received and dissected, all surviving animals were caged and observed twice a day. All animals were subjected to a detailed clinical observation before administration and such observation was conducted once every day after administration. For the general toxicity test group (groups 1-3), all surviving animals were weighed once on Day 1, 4, 7, 11, 14 and the planned autopsy time (Day 15). For the bone marrow micronucleus test group (groups 4-6), all surviving animals were weighed once on Day 1, 2 and the planned autopsy time (Day 3). Animal dose was calculated based on the latest measured body weight. For all surviving animals in the general toxicity test group (groups 1-3), the food consumption was determined once per cage on Day 2, 6, 9, 13, and calculate the average food consumption of each animal. The hematology test in the general toxicity test group (groups 1-3) was performed once at the scheduled dissection time (Day 15), and the hematology test in the bone marrow micronucleus test group (groups 4-6) was performed once at the scheduled dissection time either (Day 3). All surviving animals were fasted overnight (about 18 hours) before sample collection, and water was not allowed. Before the planned dissection, the animals were punctured through the abdominal aorta to collect blood samples for hematological analysis. At the end of the experiment, all surviving animals were anesthetized by intraperitoneal injection of 3% sodium pentobarbital (45 mg/kg). After blood collection, they were euthanized by bleeding from the abdominal aorta. All surviving animals in the bone marrow micronucleus test group (groups 4-6) were euthanized at the planned autopsy time (Day 3), according to the above method. Gross anatomy was observed, and tissues and organs were collected for fixed preservation. At the same time, femoral bone marrow from both sides was taken to carry out bone marrow smear and read the film after fixation and staining.

Genotoxicity assessment used an *in vitro* mammalian cell chromosome aberration test to detect whether the SGC8 aptamer could cause chromosome structure and number aberrations in cultured mammalian cells. According to the technical guidelines for the study of drug genotoxicity, the maximum concentration of soluble cytotoxic test materials should be determined according to the cytotoxicity, and the cytotoxicity at the highest concentration should be about 50%. According to the results of the preliminary experiment, 500 μg/mL was set as the highest concentration of the SGC8 aptamer in this experiment, and the following concentrations were 166.7 μg/mL, 55.6 μg/mL, and 18.5 μg/mL, respectively. Each concentration group had a negative control group (saline) and a positive control group (cyclophosphamide or mitomycin-C). Two flasks of cells in each group were used as parallel controls, and S9 was used for metabolism activation. After administration, drug incubation was stopped after 4 hours (±S9 group), and the culture was continued up to 24 hours later. After 24 hours of incubation (-S9 group), the cells were collected and prepared. The chromosomes of 300 metaphase cells were observed in each of the negative control groups and the test product group (-S9, ~24 h dose of 500 μg/mL group observed the chromosomes of 400 metaphase cells). One hundred chromosomes of each metaphase cell for the positive control group were observed, followed by calculating the chromosome aberration rate.

In order to evaluate the mutagenicity of SGC8 aptamer and predict its genetic hazards and potential carcinogenic effects, salmonella typhimurium reverse mutation was performed. Refer to the guidelines of bacterial reversion mutation test of compounds in the current Tripartite coordination guidelines of CFDA and International Coordination Conference (ICH), the maximum final concentration of the SGC8 aptamer in this test was 5000 μg/dish, and the dose gradient was set to 3 times. The final doses were 1667, 556, 185, 62, 21, 7, and 2 μg/dish, and the negative control group (saline) and positive control group (9-Aminoacridine, 4-Nitro-1,2phenylenediamine, cyclophosphamide, sodium azide, methyl methanesulfonate, and 2-aminofluorene) were set. Salmonella typhimurium TA97a, TA98, TA100, TA102, and TA1535 were selected as the test strains. Each dose group had 3 plates as parallel controls. Each strain was tested under both of metabolic (+S9) and non-metabolic activation (-S9) systems. The background of the test substance was normal, and no precipitation was observed in any groups. Once the SGC8 aptamer was added, the variability of each group of strains was recorded after about 66 hours of incubation to determine the mutagenicity of the SGC8 aptamer. The results showed that the positive control product could induce obvious mutagenic results compared with the negative control product, proving that the test results were reliable.

Cytotoxicity of SGC8 and NOTA-SGC8 aptamer was carried out on HCT-116 cancer cells. The cells were grown to about 80-90% confluency in a 75 cm^2^ tissue culture flask, then treated with 0.25% Trypsin. After digestion, 10,000 cells per well were seeded into 96-well culture plates. In order to ensure cell adhesion, cells were incubated overnight at 37°C with 5% CO_2_. Based on manufacturer's instructions, CCK-8 was used to determine cell viability. After 2 and 4 hours treatment with various concentration of SGC8 and NOTA-SGC8 aptamers, the cell cultural media was removed and CCK-8 reagents were added. The absorbance readout was collected by using a BioTek plate reader (measurement wavelength: 450 nm; reference wavelength: 650 nm) and cell viability was calculated in percent units.


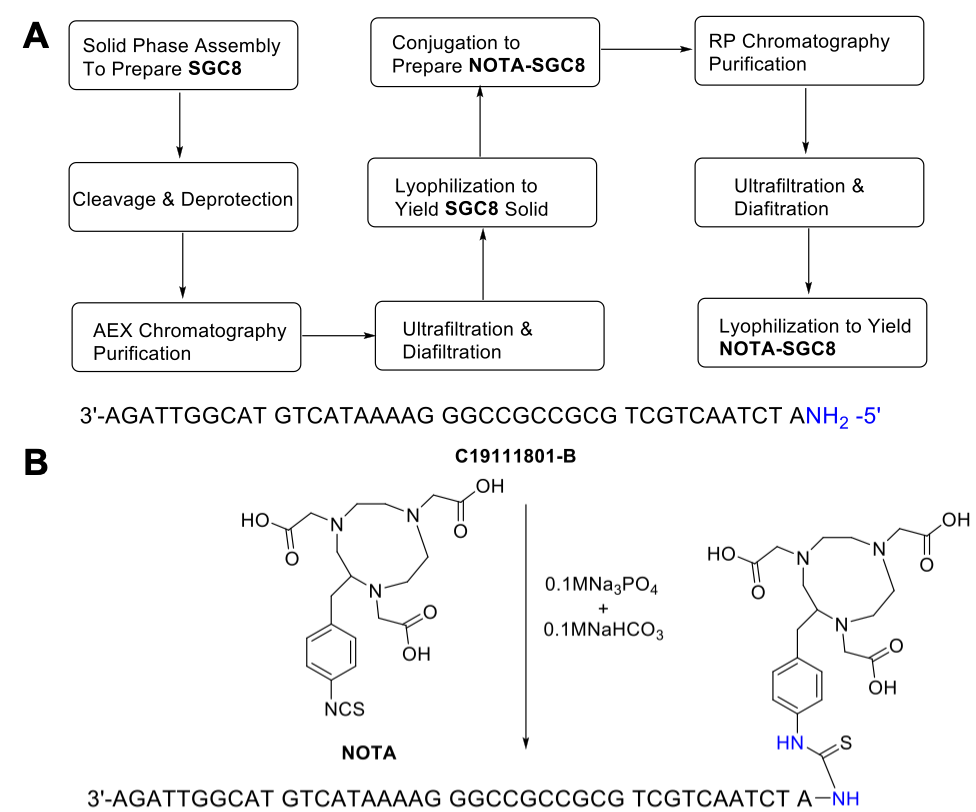


**Fig. S1. Synthesis route of NOTA-SGC8 aptamer. (A)** Synthesis procedures of NOTA-SGC8. **(B)** NH_2_-SGC8 coupling with NOTA to obtain NOTA-SGC8.

**
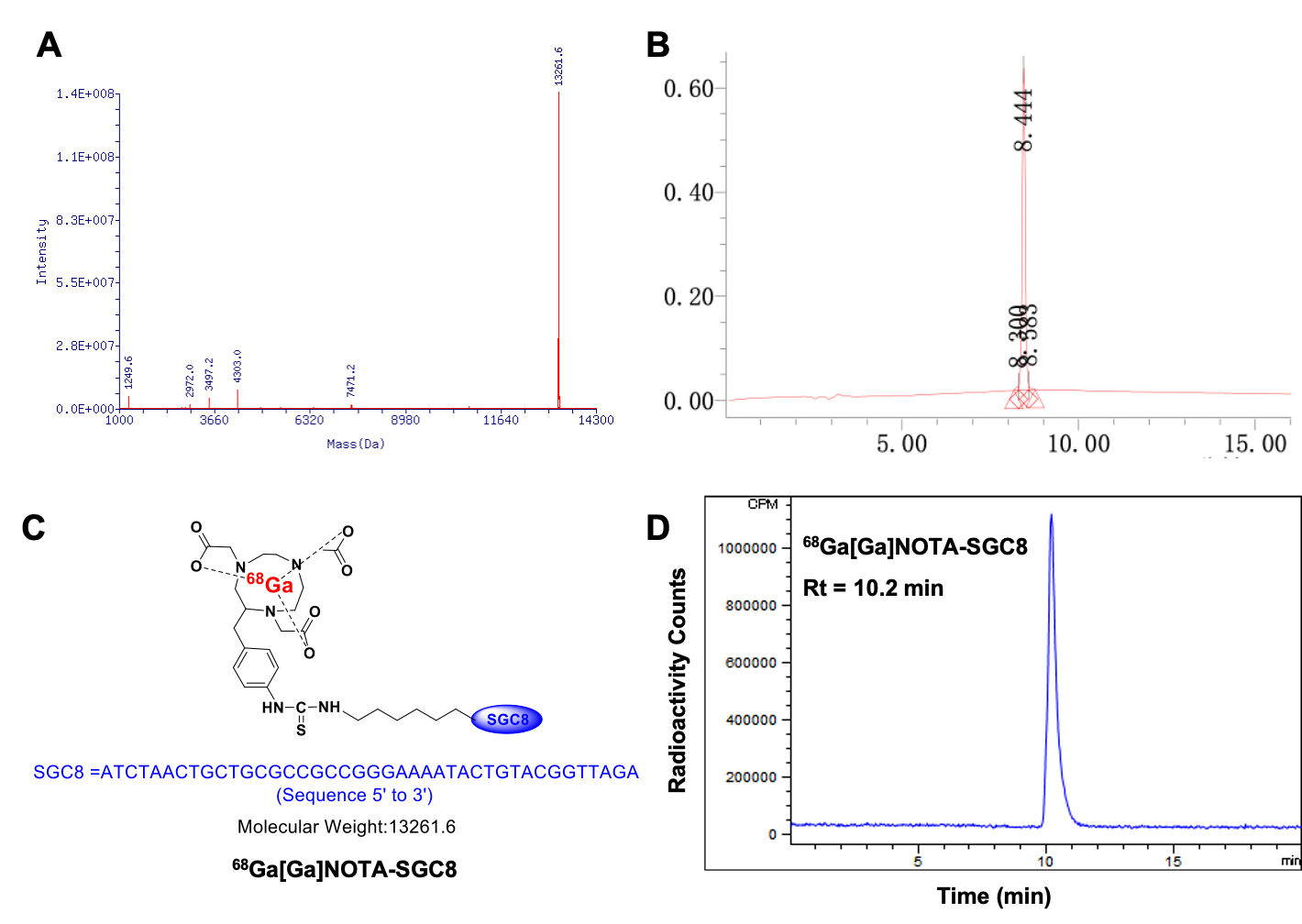
Fig. S2. Mass and HPLC analysis of NOTA-SGC8 and radiolabeled SGC8 aptamer. (A)** Mass spectrum and **(B)** HPLC confirmation of successful synthesis of NOTA-SGC8. (C) ^68^Ga[Ga]-NOTA-SGC8 structures and HPLC analysis.


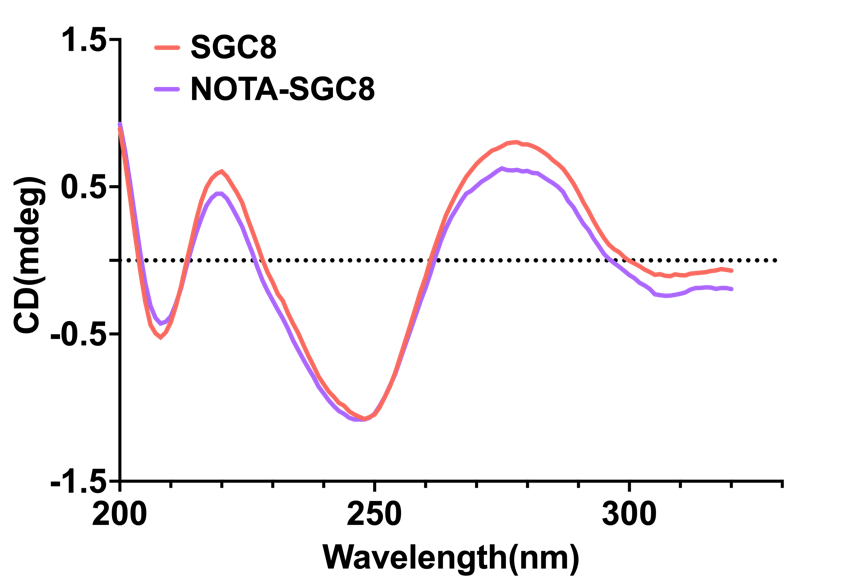


**Fig. S3. Circular dichroism spectrum characterization of SGC8 and NOTA-SGC8 aptamer.** The results indicate that the NOTA modification of SGC8 aptamer will not affect its secondary structure.


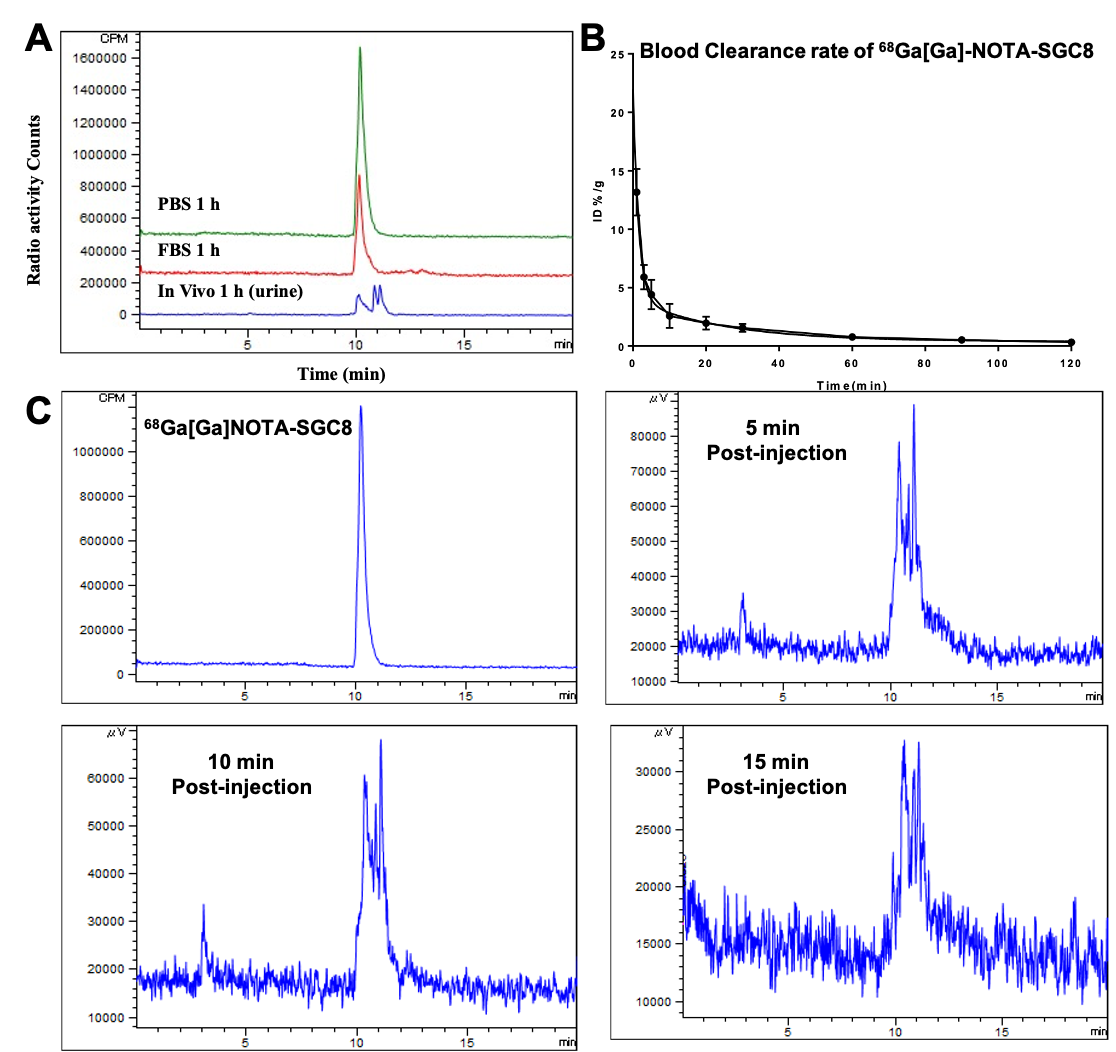


**Fig. S4. Physiological stability and pharmacokinetics investigations of ^68^Ga[Ga]-NOTA-SGC8. (A)** Radioactive HPLC study of ^68^Ga[Ga]-NOTA-SGC8 after PBS and FBS incubation, as well as urine collected from mice after i.v. administration. **(B)** Blood clearance rate of ^68^Ga[Ga]-NOTA-SGC8. **(C)** Radioactive HPLC study of ^68^Ga[Ga]-NOTA-SGC8 in mouse blood collected after multiple time points post-injection. This result showed that the radiolabeled aptamers could still be identified 15 minutes post-injection.


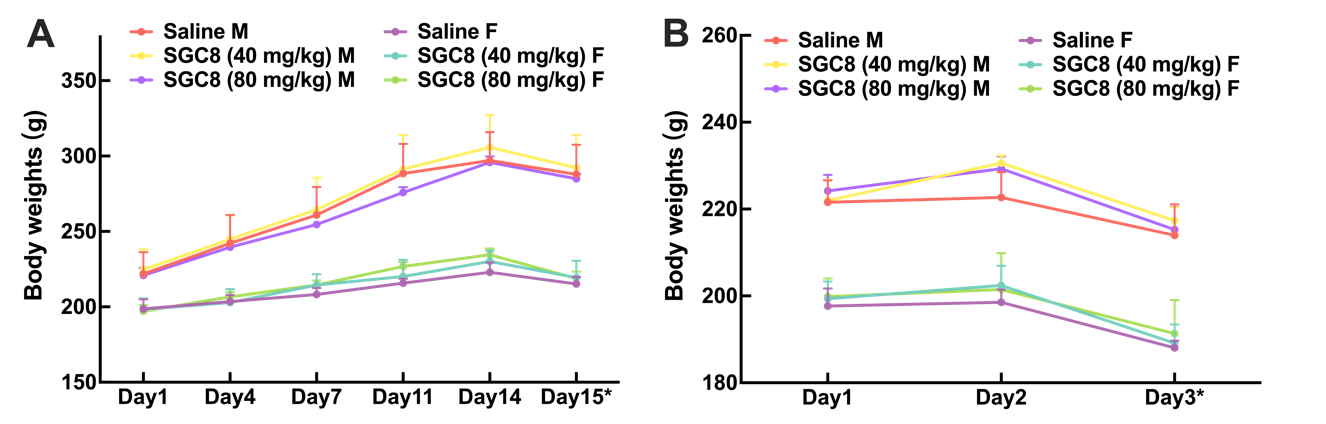


**Fig. S5. Bodyweights measurements of SD rats in single-dose toxicity (A) and mammalian erythrocyte micronucleus test (B).** All animals were grouped into saline, SGC8 (40 mg/kg), and SGC8 (80 mg/kg), each group has three male (M) and female (F) rats. *Labeled time points denote fasting treatment 24 hours before recording.


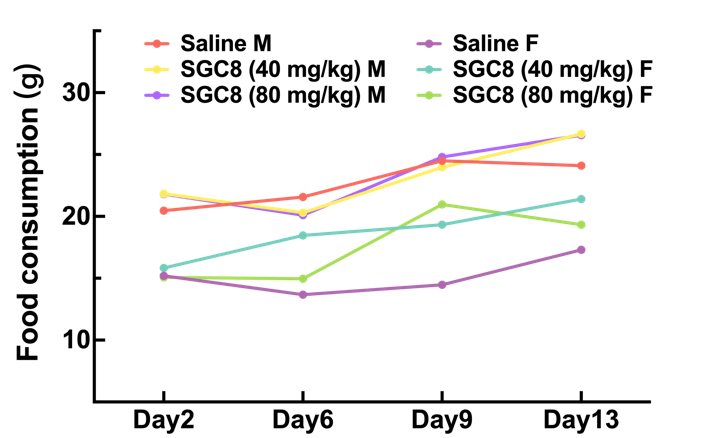


**Fig. S6. Food consumption measurements of SD rats in single-dose toxicity study.** All animals were grouped into saline, SGC8 (40 mg/kg), and SGC8 (80 mg/kg), each group has three male (M) and female (F) rats.


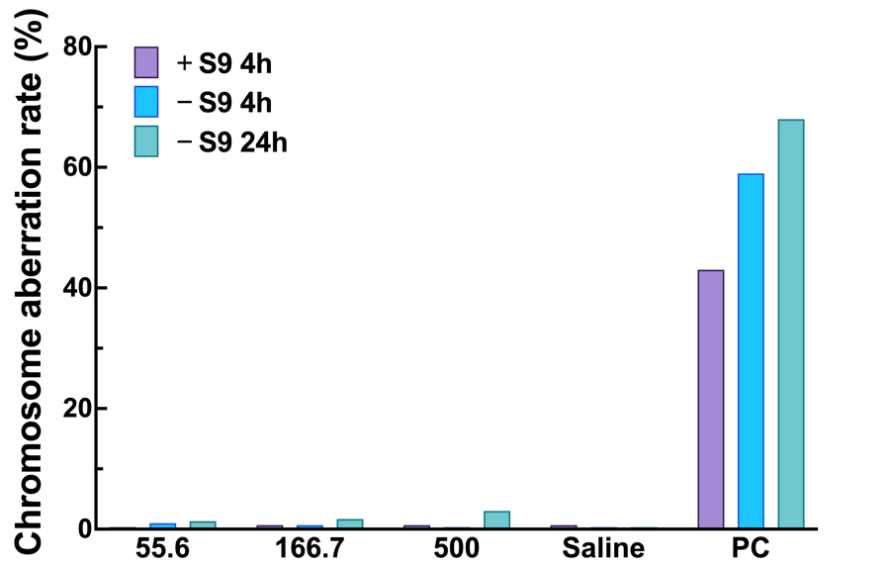


**Fig. S7. Mammalian cell chromosome aberration test of SGC8 aptamer for genotoxicity evaluation.** The highest dose of SGC8 aptamer was 500 μg/mL, and the following doses were divided into 166.7 μg/mL, 55.6 μg/mL, and 18.5 μg/mL. In each dose group, saline was set to be the negative control, cyclophosphamide (4 mg/mL), and mitomycin-C (0.05 mg/mL) were set to be positive control. Despite under the S9 metabolic activation system or not, there was no significant increase in chromosome aberration rate in each dose group (0.33%≤ aberration rate ≤3.00%), and no significant difference compared with negative control group (P > 0.05).


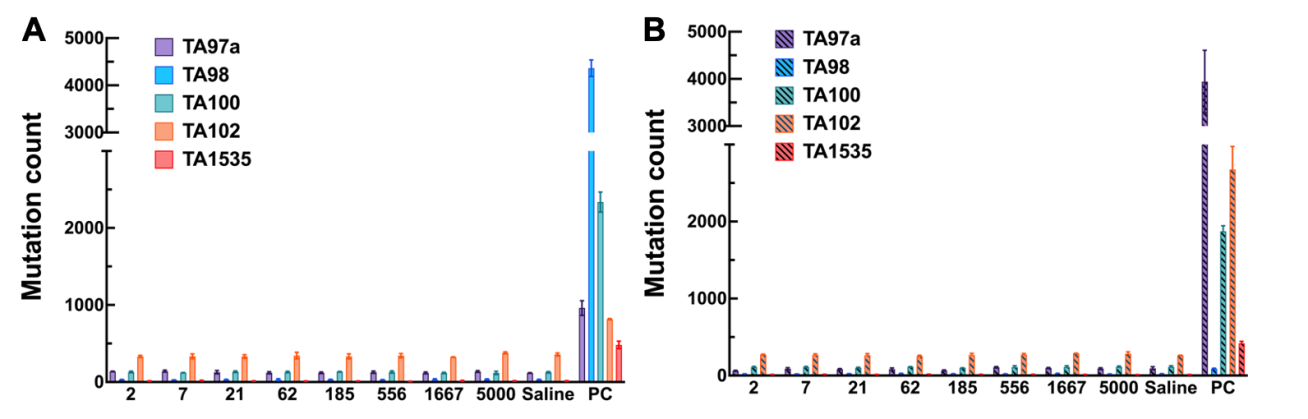


**Fig. S8. Salmonella typhimurium reverse mutation test of SGC8 aptamer for mutagenicity evaluation. (A)** With S9 and **(B)** without metabolism activation. TA97a, TA98, TA100, TA102, TA1535 were selected as the test strains. The highest dose of SGC8 aptamer was 5000 μg/dish, and the following doses were divided into 1667 μg/dish, 556 μg/dish, 185 μg/dish, 62 μg/dish, 21 μg/dish, 7 μg/dish, and 2 μg/dish. In each dose group, saline was set to be the negative control, 9-Aminoacridine (250 μg/mL), 4-Nitro-1,2phenylenediamine (40 μg/mL), cyclophosphamide (2 mg/mL), sodium azide (0.02 mg/mL), methyl methanesulfonate (10 μg/mL), and 2-aminofluorene (0.2 mg/mL, 2 mg/mL) were set to be positive control. The average number of retrograde bacteria in each dose group was less than 2 times of that in the negative control group, and there was no dose-dependent increasing relationship, so the judgment result was negative.


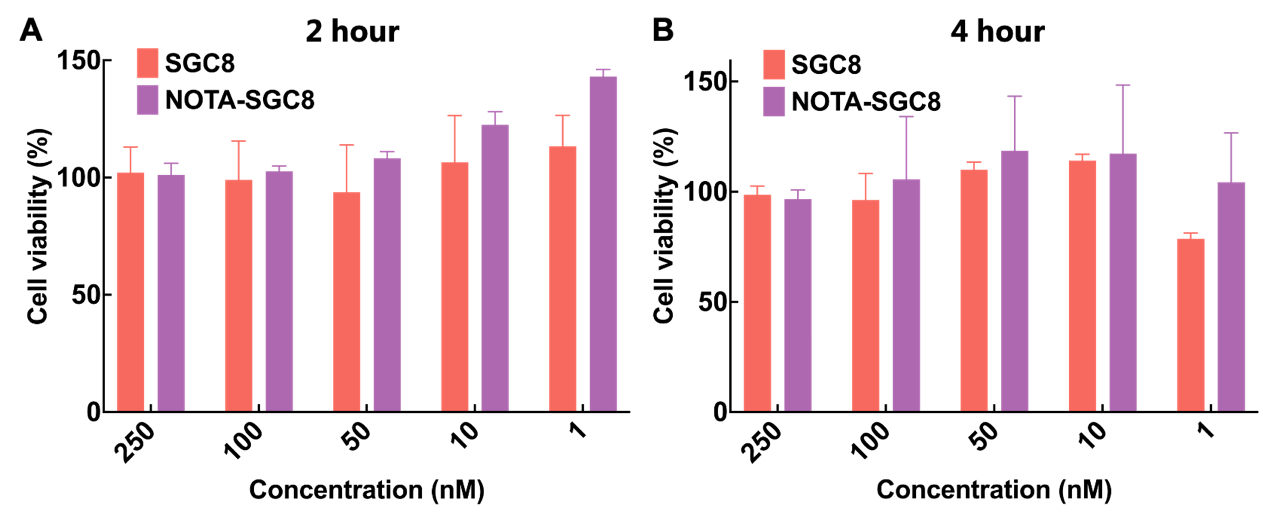


**Fig. S9. Cytotoxicity investigation of SGC8 and NOTA-SGC8 aptamer.** Both of the SGC8 and NOTA-SGC8 aptamer were incubated with HCT-116 cancer line for **(A)** 2 hour and **(B)** 4 hour.


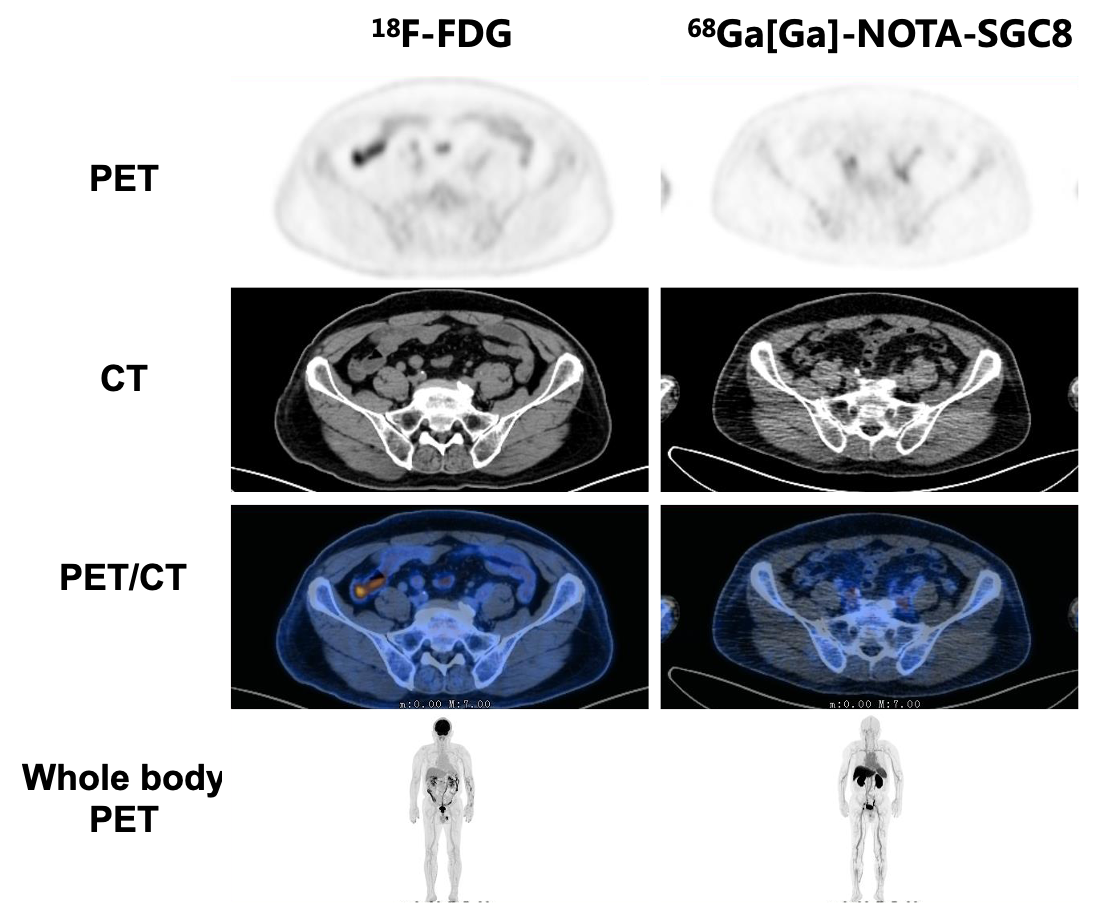


**Fig. S10. ^68^Ga[Ga]-NOTA-SGC8 and ^18^F-FDG PET/CT imaging of patient No.4.** The distribution patterns of ^68^Ga[Ga]-NOTA-SGC8 and ^18^F-FDG are totally different. Most of the ^18^F-FDG tracer is accumulated in the brain and intestine, but the ^68^Ga[Ga]-NOTA-SGC8 is accumulated in the liver and cleared through the kidney.

**
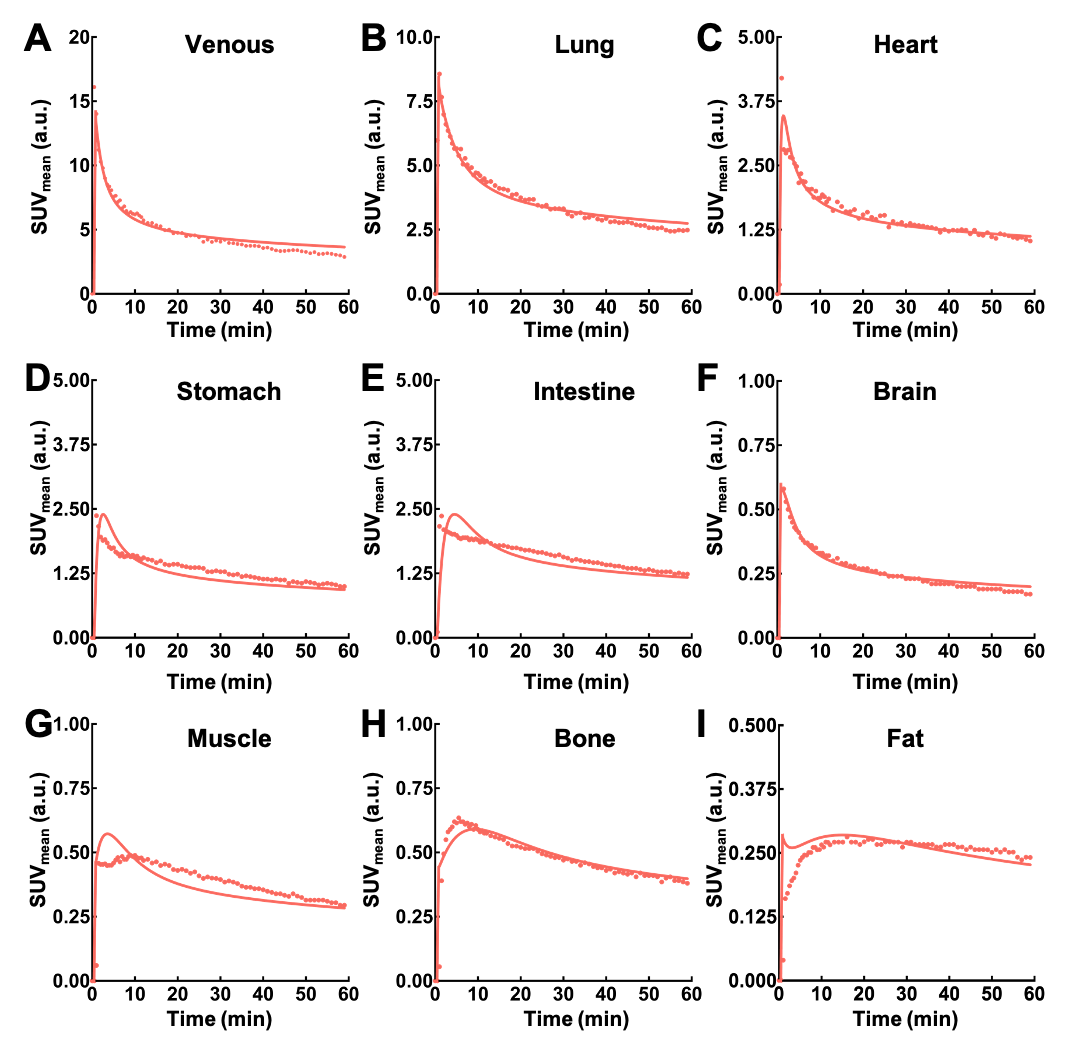
**

**Fig. S11. Whole-body PBPK model fit PK profiles of other tissues**. PBPK model predicted PK (Line) and clinical acquired PK (Dot). The fitting results showed good consistency with the clinically collected data.

**Table S1. Hematology analysis of the SD rats in the single-dose toxicity investigation.** Comparing with the saline group, SGC8 injected groups have no significant differences which mean the under the experimental dosage, SGC8 aptamer will not cause any hematology changes. White blood cell (WBC), Neutrophil (absolute count, NEUT; percentage, %NEUT), Lymphocyte (absolute count, LYM; percentage, %LYM), Monocyte (absolute count, MONO; percentage, %MONO), Eosinophil (absolute count, EOS; percentage, %EOS), Basophil (absolute count, BASO; percentage, %BASO), Red blood cell (RBC), Reticulocyte (absolute count, RET; percentage, % RET), Hemoglobin (HGB), Hematocrit (HCT), Mean red blood cell hemoglobin (MCH), mean red blood cell volume (MCV), Platelet (PLT), and mean red blood cell hemoglobin concentration (MCHC) were measured in the hematology analysis.

|  |  | **WBC (10^9^/L)** | **RBC (10^12^/L)** | **HGB (g/L)** | **HCT (%)** | **MCV (fL)** | **MCH (pg)** | **MCHC (g/L)** | **PLT (10^9^/L)** | **%RET (%)** | **RET (10^9^/L)** |
| --- | --- | --- | --- | --- | --- | --- | --- | --- | --- | --- | --- |
| Male | Saline | 8.38±2.71 | 7.5±0.68 | 150±9.17 | 41.87±2.15 | 55.97±2.71 | 20±0.85 | 358±4 | 859.33±675.66 | 7.36±0.92 | 548.5±25.32 |
|  | SGC8 (40 mg/kg) | 8.15±1.7 | 7.7±0.07 | 147.67±4.04 | 40.97±0.65 | 53.23±1.27 | 19.17±0.65 | 360.33±4.16 | 1250.67±154.02 | 7.21±0.71 | 555±51.07 |
|  | SGC8 (80 mg/kg) | 8.83±1.67 | 7.3±0.2 | 143.67±5.86 | 40.1±1.65 | 54.97±1.55 | 19.7±0.4 | 358.33±4.04 | 1287±191.56 | 7.32±0.83 | 534.7±73.42 |
| Female | Saline | 5.45±0.98 | 7.5±0.09 | 145±2.65 | 39.23±0.55 | 52.33±0.32 | 19.37±0.21 | 369.67±2.08 | 1275±49.43 | 4.8±0.76 | 359.8±53.94 |
|  | SGC8 (40 mg/kg) | 4.91±1.13 | 7.08±0.36 | 133±6.08 | 36.73±1.52 | 51.93±1.82 | 18.8±0.4 | 362.33±4.93 | 1130.33±117.74 | 6.61±1.78 | 463.73±102.25 |
|  | SGC8 (80 mg/kg) | 5.78±1.91 | 7.08±0.59 | 139±8.89 | 38.17±2.48 | 54±2.42 | 19.63±0.72 | 364±3.61 | 1324.67±108.52 | 5.85±0.53 | 413.27±43.39 |
|  |  | **NEUT (10^9^/L)** | **%NEUT (%)** | **LYMPH (10^9^/L)** | **%LYMPH (%)** | **MONO (10^9^/L)** | **%MONO (%)** | **EOS (10^9^/L)** | **%EOS (%)** | **BASO (10^9^/L)** | **%BASO (%)** |
| Male | Saline | 0.85±0.23 | 10.47±2.41 | 7.06±2.37 | 83.9±1.9 | 0.4±0.14 | 4.77±0.68 | 0.08±0.07 | 0.8±0.6 | 0.01±0.01 | 0.07±0.06 |
|  | SGC8 (40 mg/kg) | 0.96±0.27 | 12.07±3.11 | 6.73±1.51 | 82.43±3.35 | 0.4±0.13 | 4.83±0.76 | 0.05±0.02 | 0.63±0.15 | 0±0.01 | 0.03±0.06 |
|  | SGC8 (80 mg/kg) | 0.86±0.07 | 9.97±2.04 | 7.41±1.54 | 83.67±1.8 | 0.49±0.11 | 5.5±0.36 | 0.07±0.02 | 0.8±0.1 | 0.01±0.01 | 0.07±0.06 |
| Female | Saline | 0.56±0.24 | 10.1±2.63 | 4.62±0.69 | 85.03±2.86 | 0.22±0.05 | 3.93±0.29 | 0.05±0.02 | 0.93±0.25 | 0±0 | 0±0 |
|  | SGC8 (40 mg/kg) | 0.63±0.26 | 12.97±5.5 | 4.05±1.06 | 82.23±4.75 | 0.18±0.02 | 3.73±0.68 | 0.05±0.03 | 1.07±0.7 | 0±0 | 0±0 |
|  | SGC8 (80 mg/kg) | 0.63±0.24 | 10.87±0.71 | 4.85±1.58 | 83.97±1.15 | 0.23±0.09 | 3.9±0.82 | 0.07±0.02 | 1.27±0.29 | 0±0 | 0±0 |

**Table S2. Hematology analysis of the SD rats in the mammalian erythrocyte micronucleus test.** Comparing with the saline group, SGC8 injected groups have no significant differences which mean the under the experimental dosage, SGC8 aptamer will not cause any hematology changes. White blood cell (WBC), Neutrophil (absolute count, NEUT; percentage, %NEUT), Lymphocyte (absolute count, LYM; percentage, %LYM), Monocyte (absolute count, MONO; percentage, %MONO), Eosinophil (absolute count, EOS; percentage, %EOS), Basophil (absolute count, BASO; percentage, %BASO), Red blood cell (RBC), Reticulocyte (absolute count, RET; percentage, % RET), Hemoglobin (HGB), Hematocrit (HCT), Mean red blood cell hemoglobin (MCH), mean red blood cell volume (MCV), Platelet (PLT), and mean red blood cell hemoglobin concentration (MCHC) were measured in the hematology analysis.

|  |  | **WBC (10^9^/L)** | **RBC (10^12^/L)** | **HGB (g/L)** | **HCT (%)** | **MCV (fL)** | **MCH (pg)** | **MCHC (g/L)** | **PLT (10^9^/L)** | **%RET (%)** | **RET (10^9^/L)** |
| --- | --- | --- | --- | --- | --- | --- | --- | --- | --- | --- | --- |
| Male | Saline | 6.32±2.33 | 7.1±0.17 | 146±5.29 | 41.37±1.27 | 58.23±1.54 | 20.57±0.64 | 352.67±2.08 | 1299±68.02 | 8.51±1.63 | 602.97±103.19 |
|  | SGC8 (40 mg/kg) | 6.25±0.8 | 6.84±0.21 | 140.67±5.51 | 40.53±1.11 | 59.27±0.55 | 20.57±0.21 | 347±4.36 | 1209.33±23.76 | 8.89±0.86 | 607.63±59.47 |
|  | SGC8 (80 mg/kg) | 7.12±1.53 | 7.02±0.07 | 140.67±1.53 | 40.77±0.47 | 58.1±1.08 | 20.07±0.29 | 345±1.73 | 1255±77.94 | 9.22±1.57 | 646.8±104.29 |
| Female | Saline | 3.78±0.97 | 7.08±0.2 | 141±2 | 39.03±0.42 | 55.13±1.04 | 19.93±0.25 | 361±1.73 | 1259.33±87.51 | 4.13±0.33 | 292.7±28.2 |
|  | SGC8 (40 mg/kg) | 4.16±1.1 | 7.37±0.61 | 145.67±9.87 | 40.5±2.01 | 55.03±1.9 | 19.8±0.44 | 359.33±6.66 | 1225.67±159.34 | 4.07±0.65 | 298.2±30.42 |
|  | SGC8 (80 mg/kg) | 3.16±0.58 | 6.92±0.62 | 138±8.72 | 38.33±2 | 55.57±2.63 | 20±0.89 | 360±4.58 | 1340±47.29 | 3.7±0.6 | 254.33±31.12 |
|  |  | **NEUT (10^9^/L)** | **%NEUT (%)** | **LYMPH (10^9^/L)** | **%LYMPH (%)** | **MONO (10^9^/L)** | **%MONO (%)** | **EOS (10^9^/L)** | **%EOS (%)** | **BASO (10^9^/L)** | **%BASO (%)** |
| Male | Saline | 0.81±0.19 | 13.27±2.31 | 5.26±2.13 | 82.5±2.91 | 0.21±0.07 | 3.5±1.45 | 0.04±0.01 | 0.67±0.06 | 0±0.01 | 0.07±0.12 |
|  | SGC8 (40 mg/kg) | 0.63±0.13 | 9.97±1.14 | 5.31±0.72 | 84.97±1.97 | 0.26±0.08 | 4.23±1.45 | 0.05±0.01 | 0.77±0.12 | 0±0.01 | 0.07±0.12 |
|  | SGC8 (80 mg/kg) | 0.92±0.53 | 12.37±4.42 | 5.89±0.9 | 83.37±5.07 | 0.26±0.12 | 3.5±0.78 | 0.04±0.01 | 0.63±0.15 | 0.01±0.01 | 0.13±0.12 |
| Female | Saline | 0.33±0.11 | 8.87±2.66 | 3.28±0.85 | 86.87±2.76 | 0.11±0.04 | 2.9±0.26 | 0.05±0.02 | 1.37±0.21 | 0±0 | 0±0 |
|  | SGC8 (40 mg/kg) | 0.4±0.19 | 9.3±2.03 | 3.53±0.93 | 84.83±2.58 | 0.16±0.02 | 4.1±1.13 | 0.07±0.02 | 1.77±0.74 | 0±0 | 0±0 |
|  | SGC8 (80 mg/kg) | 0.38±0.16 | 11.97±4.45 | 2.6±0.48 | 82.5±4.43 | 0.14±0.03 | 4.5±0.47 | 0.03±0.02 | 0.9±0.56 | 0±0.01 | 0.1±0.17 |

**Table S3. Genotoxicity analysis of SGC8 aptamers by bone marrow micronucleus test in the SD rats.** Polychromatic erythrocytes (PCE) and normochromatic erythrocyte (NCE) were recorded. Comparing with the saline group, SGC8 (40 mg/kg) injected group have no significant differences. The SGC8 (80 mg/kg) group’s micronucleus rate was significant different from saline group.

|  |  | **PCE total (PCS)** | **PCE with micronucleus (PCS)** | **PCE^#^ (PCS)** | **NCE^&^ (PCS)** | **PCE/(PCE+NCE)（%）** | **Micronucleus rate (‰)** |
| --- | --- | --- | --- | --- | --- | --- | --- |
| Male | Saline | 12033 | 19 | 819 | 681 | 54.60 | 1.58 |
|  | SGC8 (40 mg/kg) | 12057 | 26 | 719 | 781 | 47.93 | 2.16 |
|  | SGC8 (80 mg/kg) | 12401 | 46 | 782 | 727 | 51.82 | 3.71* |
| Female | Saline | 12239 | 21 | 776 | 728 | 51.60 | 1.72 |
|  | SGC8 (40 mg/kg) | 12148 | 27 | 644 | 858 | 42.88 | 2.22 |
|  | SGC8 (80 mg/kg) | 12082 | 56 | 766 | 736 | 51.00 | 4.63* |
| *Chi-square test was used for statistical comparison, n=3, P ≤ 0.05;  ^#^ PCE, the number of PCE in at least 500 red blood cells (PCE+NCE) counted per animal;  ^&^ NCE, the number of NCE in at least 500 red blood cells (PCE+NCE) counted per animal. | | | | | | | |

**Table S4. Patient characteristics in aptamer first-in-human dynamic Pharmacokinetics study.**

| **Patient No.** | **Gender** | **Age** | **Tumor Types** | **Body weights (Kg)** | **Injected dose (mCi)** |
| --- | --- | --- | --- | --- | --- |
| 1 | Female | 37 | Breast cancer after surgically excision | 64 | 2.94 |
| 2 | Male | 42 | Nasopharyngeal carcinoma | 72 | 2.99 |
| 3 | Female | 65 | Ovarian cancer after surgically excision | 69 | 3.36 |
| 4 | Male | 63 | Colorectal cancer | 71 | 5.84 |

**Table S5. Human (male, 63 years old, 71 kg) physiological parameters used to build the whole-body PBPK model and the delineated methods of each VOI.**

|  | **Total volume (kg)** | **Vascular volume (kg) *V_v,i_*** | **Extravascular volume (kg) *V_e,i_*** | **Blood flow (L/min) *Q_i_*** | | **Delineated**  **method** |
| --- | --- | --- | --- | --- | --- | --- |
| **Aorta** | 0.35 | 0.3500 | - | 5.66 | Left ventricle | |
| **Venous** | 0.79 | 0.7900 | - | 5.66 | Right ventricle | |
| **Lung** | 0.80 | 0.4640 | 0.3360 | 5.66 | Full organ^b^ | |
| **Brain** | 1.54 | 0.0616 | 1.4784 | 0.71 | Full organ | |
| **Heart** | 0.41 | 0.0574 | 0.3526 | 0.23 | Heart wall | |
| **Liver** | 2.09 | 0.3553 | 1.7347 | 0.37 | Full organ | |
| **Spleen** | 0.15 | 0.0495 | 0.1005 | 0.15 | Full organ | |
| **Stomach** | 0.15 | 0.0045 | 0.1455 | 0.06 | Full organ^b^ | |
| **Intestine** | 1.01 | 0.0202 | 0.9898 | 0.8 | Full organ^b^ | |
| **Kidney** | 0.41 | 0.0943 | 0.3157 | 1.16 | Full organ | |
| **Muscle** | 27.52 | 0.8256 | 26.6944 | 0.95 | Thigh muscle | |
| **Bone** | 8.42 | 0.2526 | 8.1674 | 0.27 | femur | |
| **Fat** | 23.23 | 0.4646 | 22.7654 | 0.06 | Gluteal adipose tissue | |
| Other | 4.13 | 1.0369 | 3.0931 | 0.36 | Remaining weight | |

The density of all tissues and blood were assumed to be 1.0 kg/L. a. The physiological parameters were taken from the PK-Sim (Open Systems Pharmacology suite Version 10.0). b. For the hollow organs, include lung, stomach and intestine, the SUV_B/W_ were corrected by removing the cavity volumes.

**Table S6. PBPK model estimated parameters**

|  | ***PS_i_* (L/min)** | ***PC_i_*** |
| --- | --- | --- |
| **Lung** | 0.0106 | 0.4083 |
| **Brain** | 0.0035 | 0.0152 |
| **Heart** | 0.0286 | 0.1941 |
| **Liver** | 0.0275 | 1.0681 |
| **Spleen** | 0.001 | 0.7579 |
| **Stomach** | 0.0057 | 0.2323 |
| **Intestine** | 0.0217 | 0.3037 |
| **Kidney** | 0.0144 | 2.2492 |
| **Muscle** | 0.0807 | 0.0486 |
| **Bone** | 0.0157 | 0.0787 |
| **Adipose** | 0.0129 | 0.0405 |
| **Others** | 0.0213 | 1.7794 |
| *CL_renal_* | 0.0068 (L/min) |  |
| *f_u_* | 0.1011 |  |

*CL_renal_*, renal clearance

*f_u_*, fraction of unbound probe in whole blood

**Eq. S1-S6 Ordinary Differential Equations of whole-body PBPK model.**

**Eq. S1. Aorta**

**Eq. S2. Venous**

$$Q_{hep} =Q_{liver} +Q_{spleen} +Q_{stomach} +Q_{intestine}$$

**Eq. S3. General permeability-limited model equations**

**Eq. S4. Lung**

**Eq. S5. Kidney**

**Eq. S6. Liver**
